# Supplementary material for: Disentangling the Effects of Colocalizing Genomic Annotations to Functionally Prioritize Non-coding Variants within Complex-Trait Loci
Source: Am J Hum Genet. 2015 Jul 2;97(1):139–52. doi: 10.1016/j.ajhg.2015.05.016 (PMC4572568; doi:10.1016/j.ajhg.2015.05.016)
Supplement: Document S1. Figures S1–S8 and Tables S1 and S3 [file mmc1.pdf]

The American Journal of Human Genetics

Supplemental Data

Disentangling the Effects of Colocalizing Genomic Annotations to Functionally Prioritize Non-coding Variants within Complex-Trait Loci

Gosia Trynka, Harm-Jan Westra, Kamil Slowikowski, Xinli Hu, Han Xu, Barbara E Stranger, Robert J Klein, Buhm Han, and Soumya Raychaudhuri

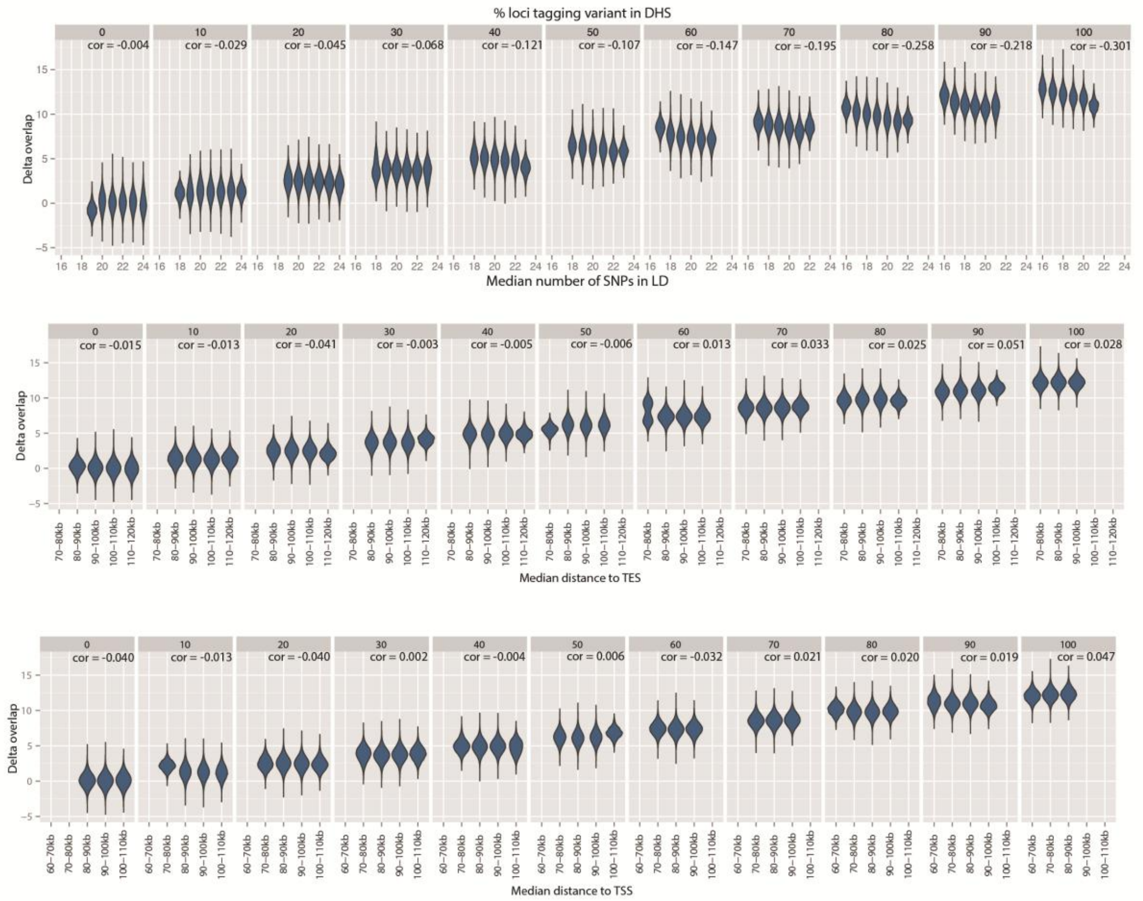

Figure S1. Influence of different genomic parameters on delta-overlap. For each of the 1,000 simulated SNP sets tagging variable percent of DHS variants, we assessed if SNP structure (measured by the number of LD SNPs) or genomic features, such as proximity to TSS and TES, could influence the estimates of delta-overlap. For each SNP set, we plot the median characteristics and the set and the delta overlap. While we observed that the proportion of causal variants within DHS regions was proportional with the delta-overlap, for a fixed proportion of causal DHS variants delta-overlap is stable for all genomic features (r^2<0.1). We observed weak correlation with the number of LD SNPs in SNP sets that were highly saturated with DHS-tagging loci (> 60%).

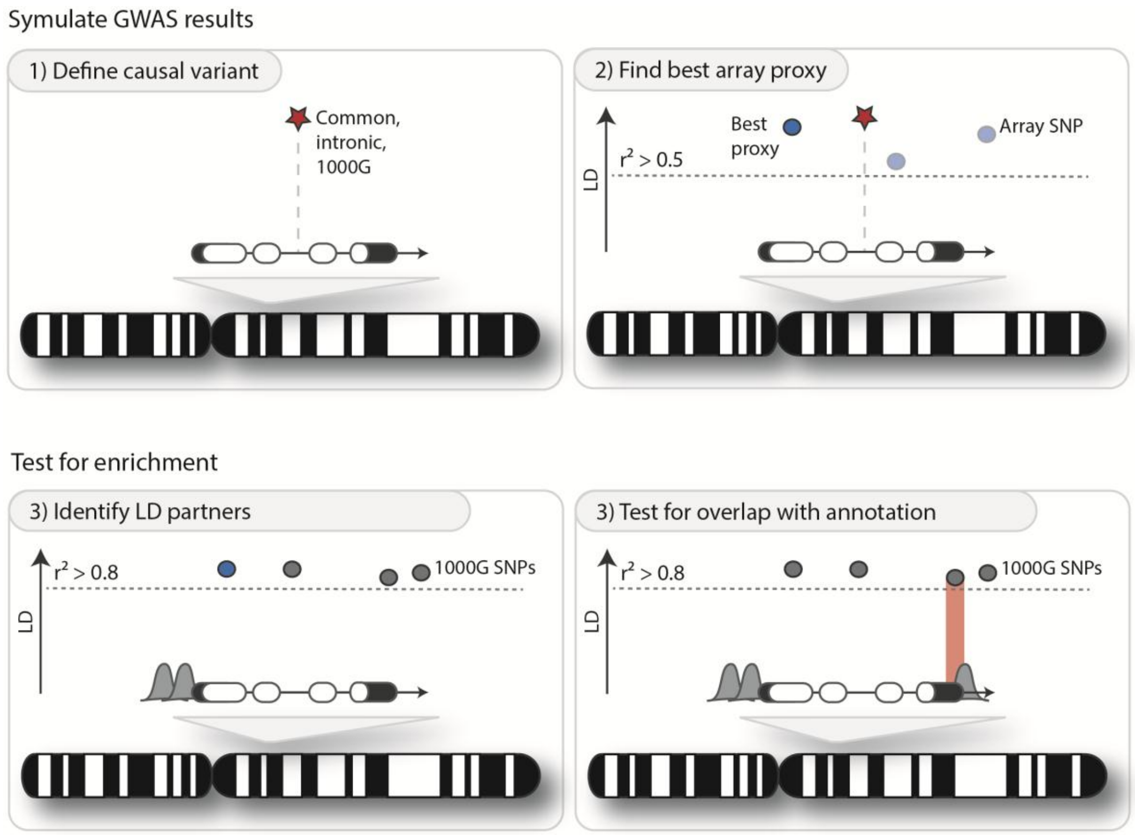

Figure S2. Schematic figure of the strategy to simulate the GWAS SNP sets. From 1000 Genomes common European variants, we pick a functional SNP that maps within a pre-defined genomic annotation, e.g. intron. To imitate a GWAS approach we then identify the SNP on the genotyping array (Illumina Human Omni2.5 chip) that best tags (r^2>0.5) the selected predefined functional variant. We construct sets of 1,416 SNPs tagging predefined functional variants. These sets are then subject to enrichment tests.

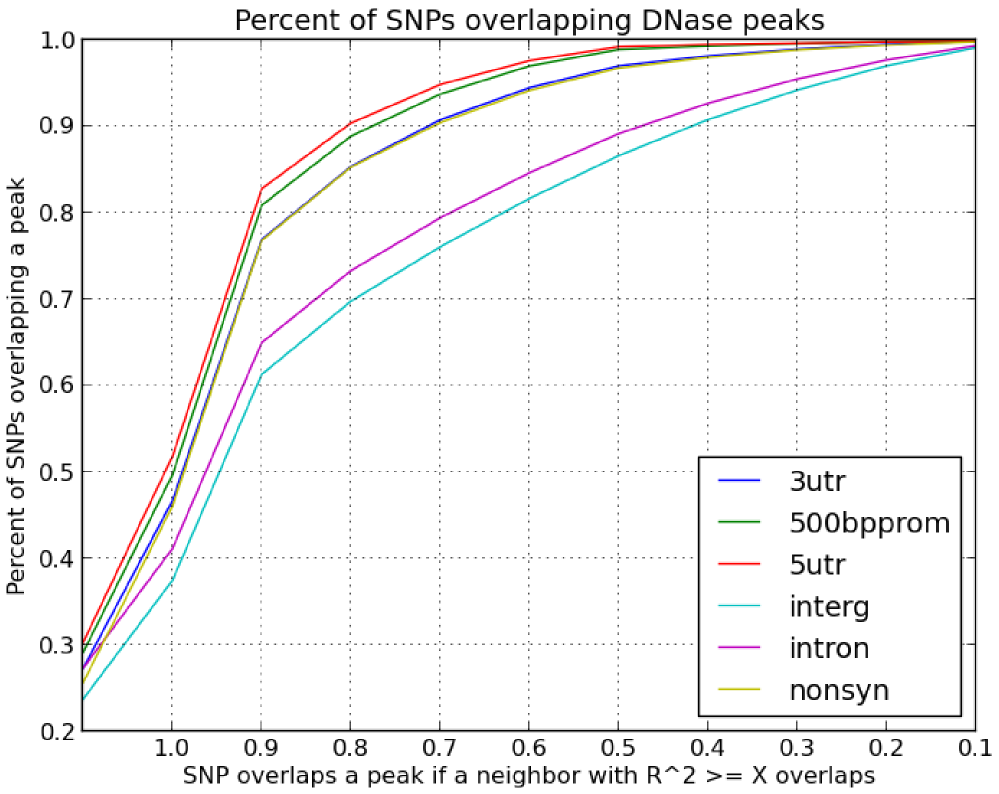

Figure S3. Quantification of measured overlap for Illumina Omni2.5 SNPs tagging functional variants from different annotations. About 30% of SNPs at Illumina Omni2.5 overlap with DHS sites on their own. This percentage quickly increases as additional linked SNPs are included. At r^2=0.8 we should observe the strongest enrichment for promoter and 5'UTR SNPs, moderate signals for coding (non-synonymous) and 3'UTR, and no enrichment for intron or intergenic regions. The decrease in r^2 threshold increases the percentage of SNPs overlapping with DHS. Importantly, when deriving the null through matching-based tests, not accounting for the number of LD SNP dramatically affects the enrichment results.

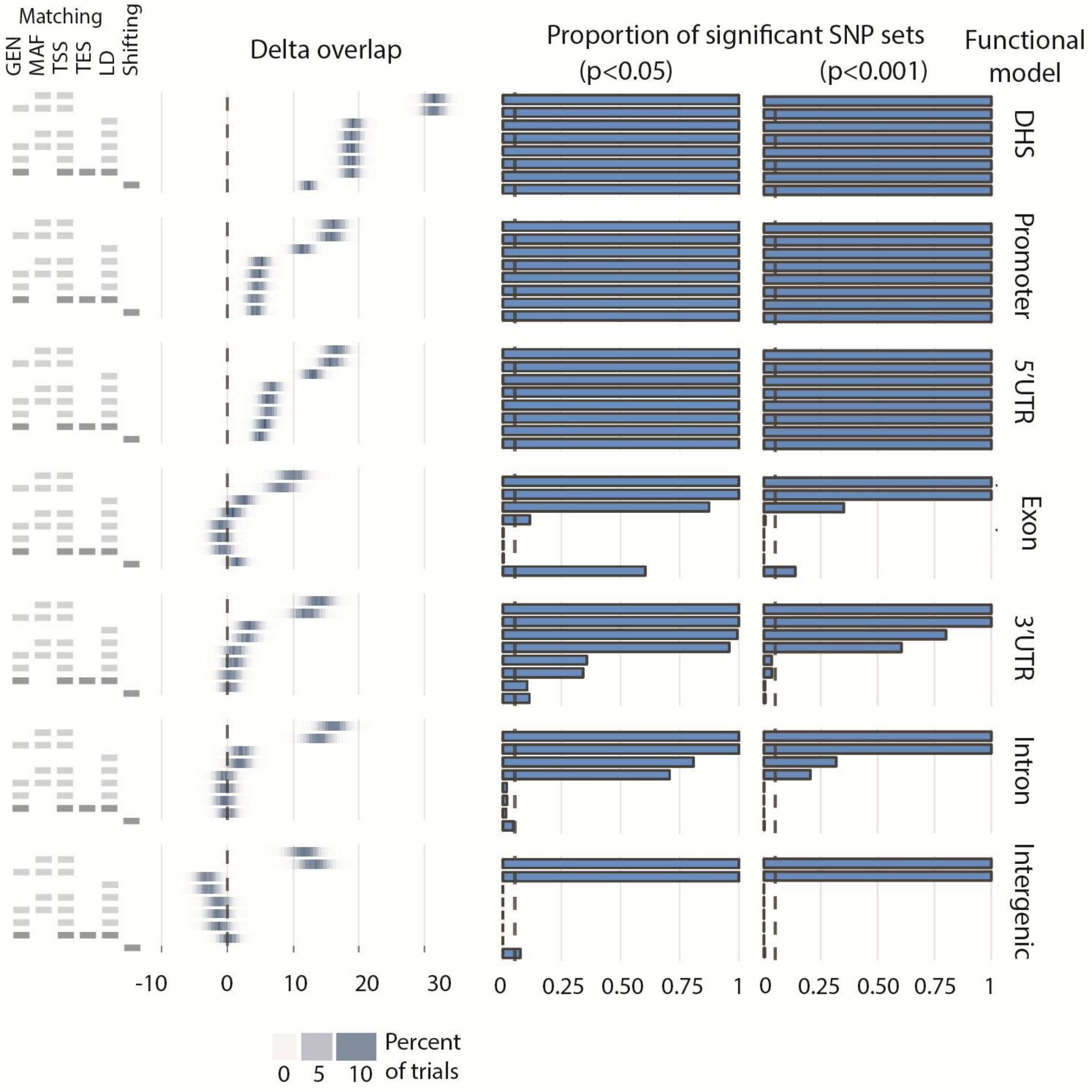

Figure S4. **Comparison of the effect sizes (delta-overlap) and power of different enrichment methods.** Not matching on the number of SNPs in LD results in global inflation in statistics. For example, we observed p<0.001 significance across all regulatory and non-regulatory SNP sets. However, matching on LD alone is insufficient; we observed consistently inflated type I error across SNP sets tagging functional variants from introns (p<0.05 in 81% of 1,000 SNP sets). We also observed that the standard matching parameters were inadequate across SNP sets that tagged variants in 3'UTR and exons. Accounting for the distance to the end of transcription (TES) was crucial if functional variants were selected from the 3'UTR, and it decreased the false positive rate by 26% (at p<0.05). Note that including MAF in the matching parameters has no effect, even though it is a frequently used parameter in SNP matching-based tests.

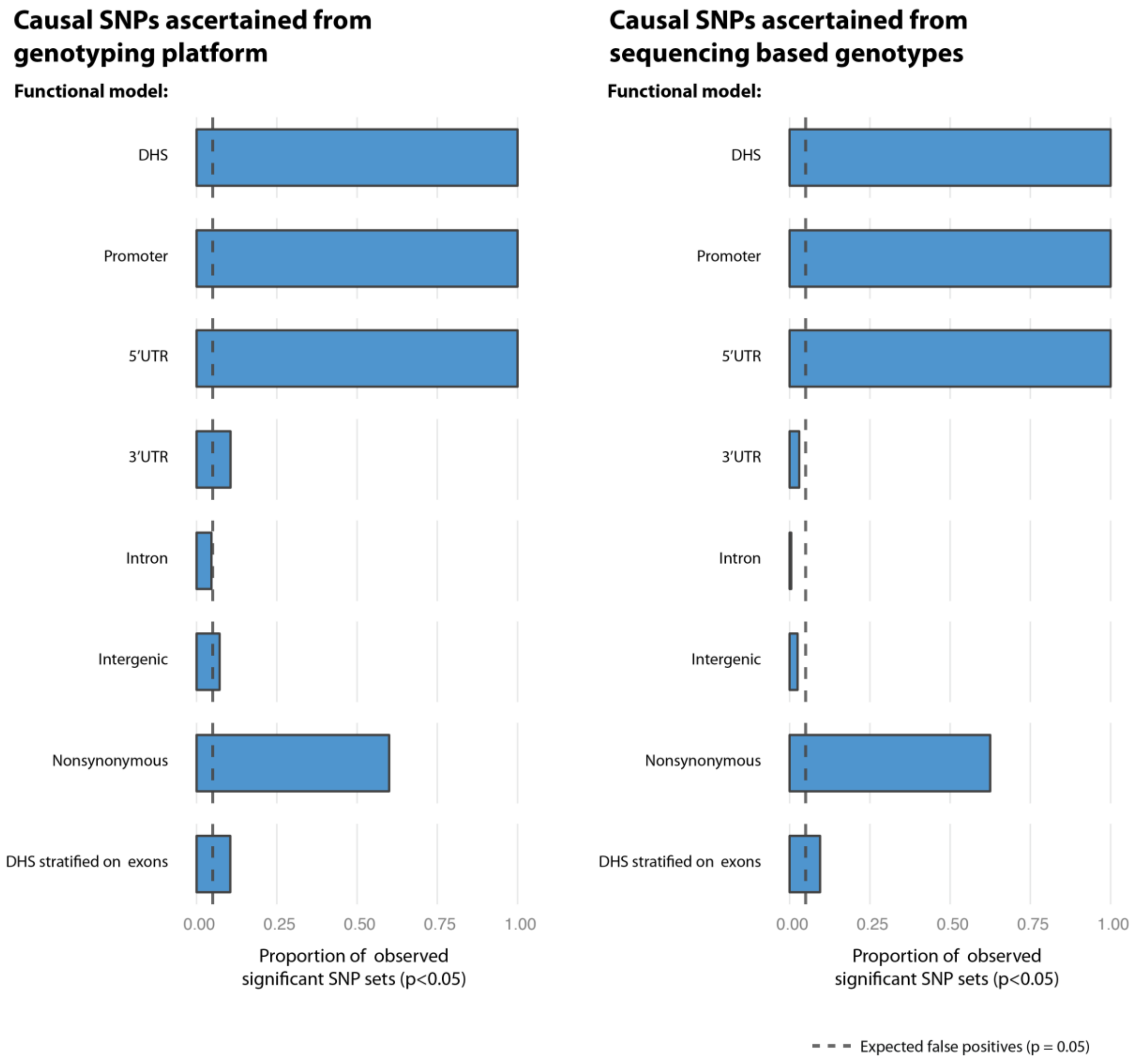

Figure S5. Performance of *GoShifter* when selecting tag variants from sequencing data. We generated sets of 1,416 SNPs that tag functional variants from SNPs within annotations enriched for DHS (SNPs overlapping DHS, promoter regions, 5' UTR, nonsynonymous variants in exons) or depleted for DHS (3'UTR, introns and intergenic regions). We generated 1,000 sets of SNPs where we selected tagging SNPs from a commercial genotyping platform (left), and 200 sets of tagging SNPs were selected from a sequencing based study (1000 Genomes Project, right). Array-based data is identical to the data presented in Figure 2. Then we subsequently tested for enrichment for DHS with GoShifter. We also tested the number of SNP sets obtaining $p<0.05$ stratifying DHS for sets where causal variants were in exons. The proportion of expected sets $p<0.05$ under the null is indicated by the dotted line.

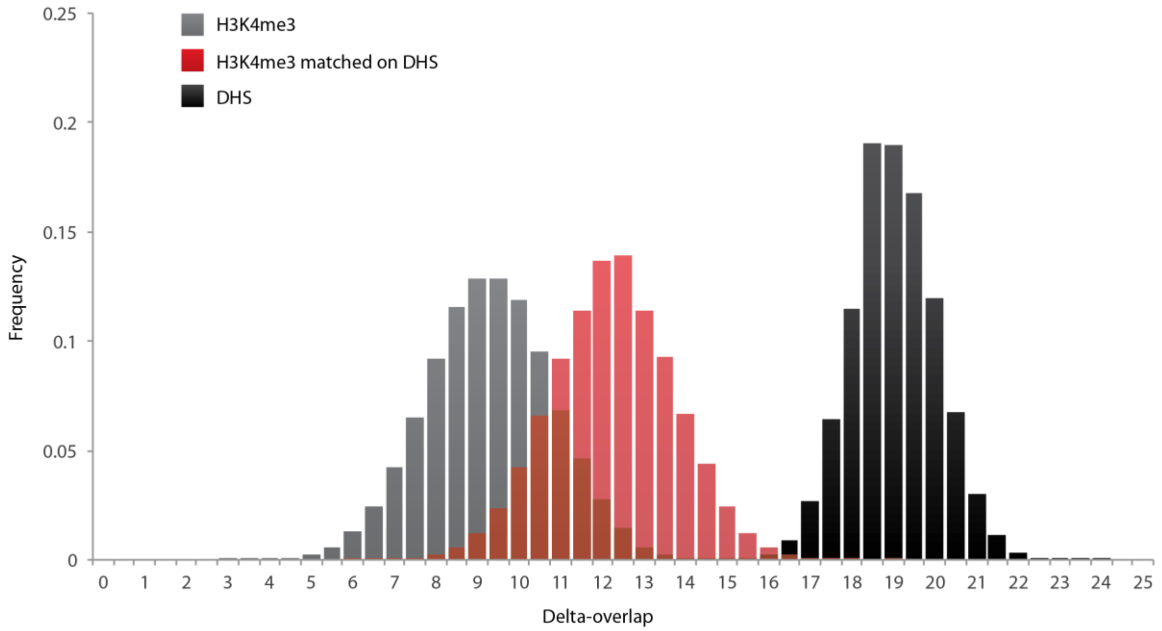

Figure S6. Conditional matching and colocalizing annotations. We assessed the feasibility of using a matching based approach to disentangle colocalizing annotations. For this purpose, we generated 1,000 sets of SNPs tagging functional variants in DHS. We tested these sets for enrichment with DHS (black) and the colocalizing annotation H3K4me3 (grey) using matching based on (GEN, TSS, TES and LD). To account for colocalization, we appended an extra matching parameter (DHS) and repeated the H3K4me3 enrichment analysis (red). We found that each of these enrichment analyses yielded significant enrichment ($p<0.05$) for 100% of the generated sets (as indicated by the delta-overlap values being all greater than zero). Matching on the additional DHS parameter did not mitigate the delta-overlap for H3K4me3 enrichment. This is probably due to confounding factors included by the extra DHS matching parameter (for example LD bias within DHS overlapping variants), and the lack of correlation between the associated and causal SNP in the annotations they occur in.

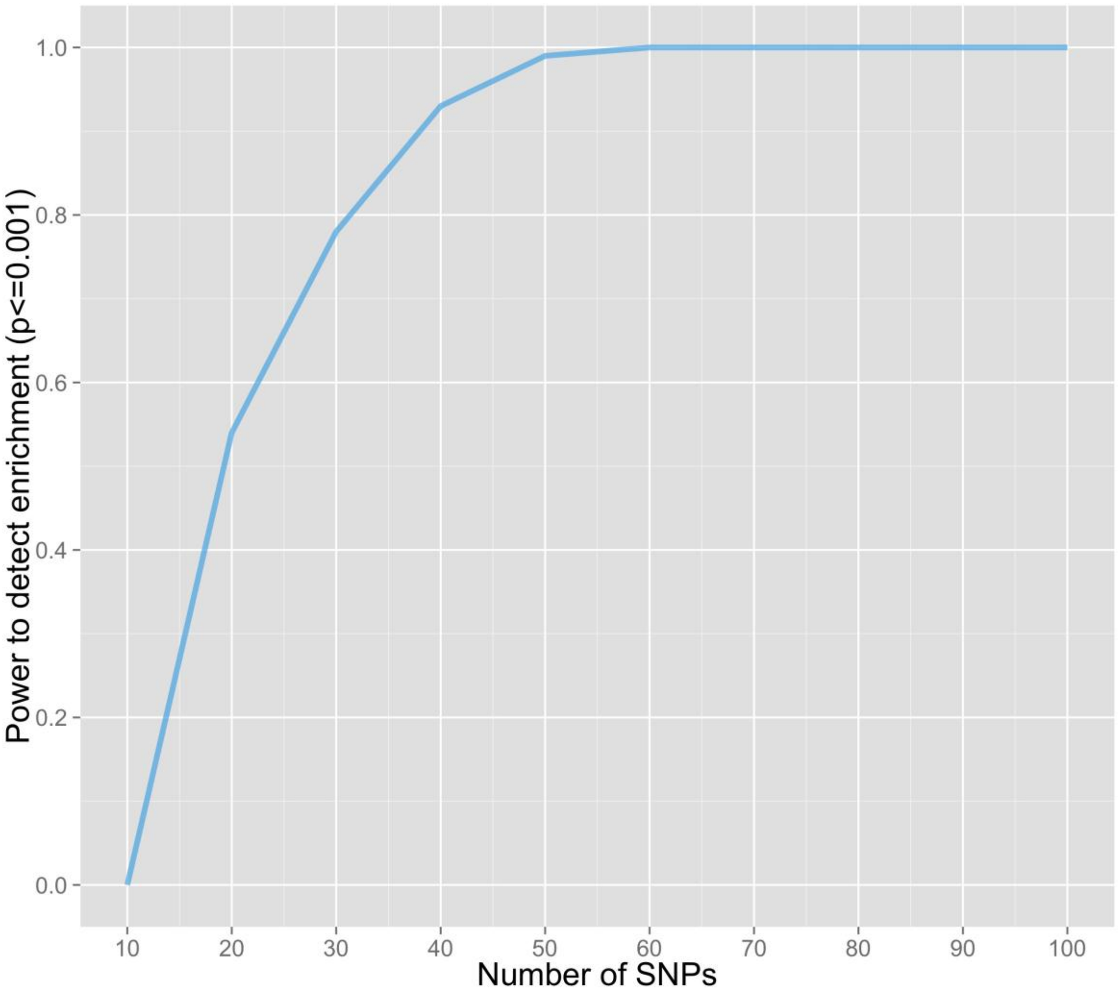

Figure S7. Power to detect significant enrichment as a function of the number of tested SNPs. We subsampled SNPs from SNP sets tagging variants in DHS regions (Figure 2A and S4 – functional model with DHS variants). To obtain power estimates for *GoShifter* to detect significant enrichment we generated a hundred SNP sets with variable number of variants (from 10 to 100 incremented by 10 SNPs). With 30 SNPs *GoShifter* has 80% of power to detect significant enrichment ($p<0.05$).

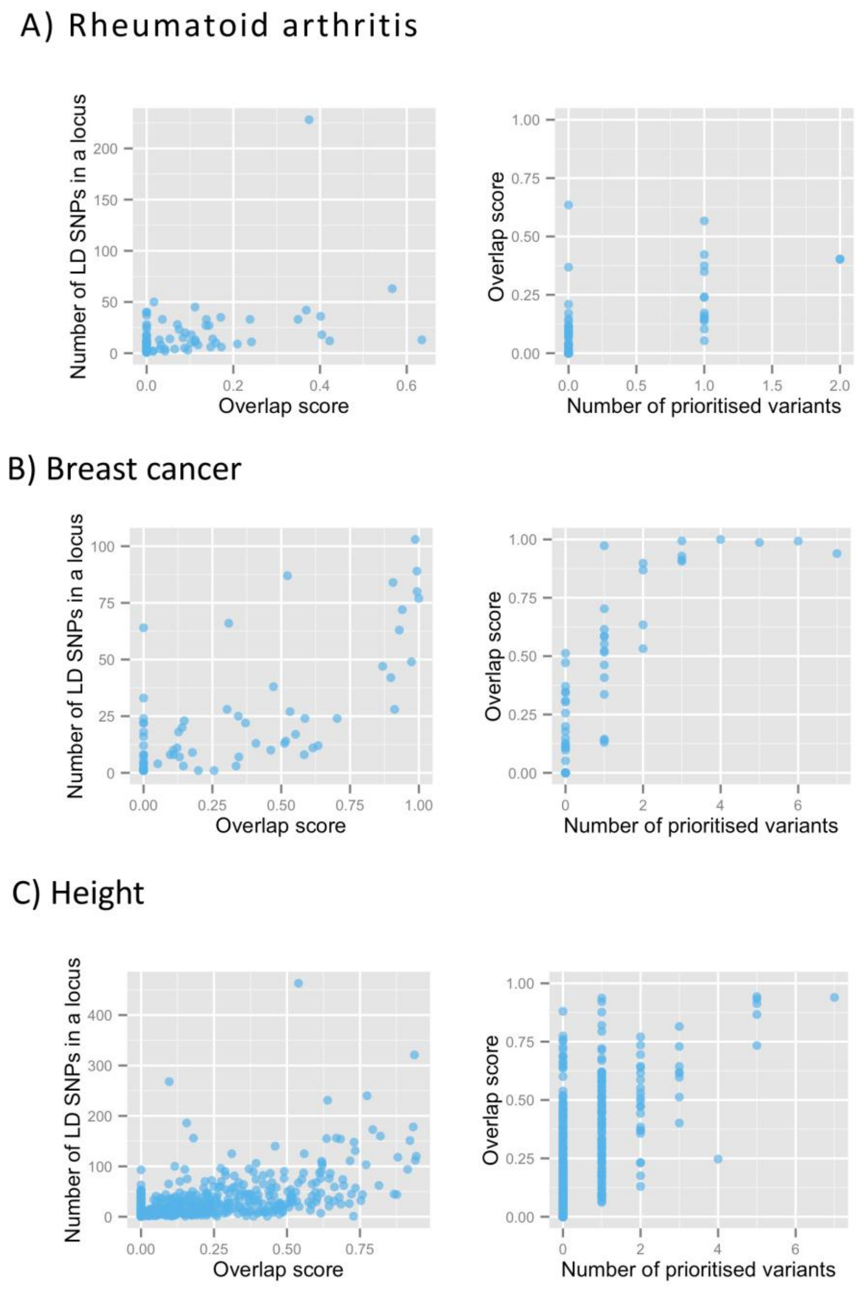

Figure S8. Prioritization of functional variants using overlap score. Here we define a prioritized variant, as a variant(s) within a locus overlapping the annotation in question. For each tested trait we plot the relationship of the overlap score of each locus and the number of SNPs in LD within each associated locus (left). We also plotted the relationship of the overlap score and the number of those SNPs in LD overlapping an annotation site (e.g. prioritized variants).

Table S1. Overview of published enrichment methods. Published statistical strategies within the literature to assess enrichment for different annotations.

Trait	Annotation	Enrichment method	Reference
NHGRI GWAS Catalog	TFs ChIP-seq, DHS	Matching on MAF, TSS, platform, UCSC gene predicted function	[1]
144 diseases	NF-kB ChIP-seq	Matching on MAF, TSS	[2]
Platelet and erythrocyte phenotypes	FAIRE-seq	Matching on MAF, TSS and LD	[3]
Breast cancer	TF and histone modification ChIP-seq	LD	[4]
Primary biliary cirrhosis	DHS, FAIRE-seq	Permutations with SNPs within associated loci	[5]
Asthma	Chromatin HMM states, H3K4me1, H3K27ac	None	[6]
NHGRI GWAS Catalog	DHS	MAF, TSS, GENIC	[7]
NHGRI GWAS Catalog	Intronic splicing enhancers	MAF, TSS	[8]
NHGRI GWAS Catalog - trans-eQTL SNPs	CNVs, TFBS, splice enhancers/silencers, histone enhancers	Relative to disease associated non-trans-eQTL SNPs	[9]
NHGRI GWAS Catalog	DHS, TFBS	Matching on MAF, TSS, genomic location	[10]
Migraine	DHS	MAF, TSS, GC content	[11]
Lipid levels	Chromatin HMM states, histone modifications, open chromatin	MAF, TSS, LD partners	[12]
eQTLs	Histone modifications	MAF, TSS	[13]
Colorectal cancer SNPs	Enhancers	LD, IlluminOmniExpress	[14]
Immune-related disease SNPs	Enhancers	LD, IlluminOmniExpress	[15]
Specific traits from GWAS Catalog; T2D and fasting glycemia	Human pancreatic islet TFBS and enhancer clusters	LD; TSS, MAF	[16]
GWAS Catalog traits	Chromatin states	Shifts and permutations within GWAS Catalog traits	[17]
Rare variants and structural variations (SVs)/SNPs/eQTLs	GENECODE annotation/functional annotations	Annotation shifting/MAF+TSS matching	[18]

Table S2. Detailed description of used cell types for DHS, H3K4me1, H3K4me3, and H3K9ac.

Table S3. Proportion of 1,000 SNP sets, derived from causal variants with specific functional annotations, demonstrating enrichment at $p<0.05$.

Enrichment method	Promoter	5'UTR	Non-synonymous	3'UTR	Intron	Intergenic
**GEN+TSS+TES+LD**	1	1	0.002	0.102	0.013	0

**GEN+MAF+TSS+LD**	1	1	0.001	0.358	0.016	0
**GEN+MAF+TSS**	1	1	1	1	1	1
**GEN+TSS+LD**	1	1	0.001	0.342	0.017	0
**MAF+TSS+LD**	1	1	0.114	0.959	0.708	0
**MAF+TSS**	1	1	1	1	1	1
**LD**	1	1	0.875	0.994	0.808	0
**Local shifts**	1	1	0.604	0.112	0.044	0.074

References

1. Schaub, M.A., Boyle, A.P., Kundaje, A., Batzoglou, S., and Snyder, M. (2012). Linking disease associations with regulatory information in the human genome. Genome research 22, 1748-1759.
2. Karczewski, K.J., Dudley, J.T., Kukurba, K.R., Chen, R., Butte, A.J., Montgomery, S.B., and Snyder, M. (2013). Systematic functional regulatory assessment of disease-associated variants. Proceedings of the National Academy of Sciences of the United States of America 110, 9607-9612.
3. Paul, D.S., Albers, C.A., Rendon, A., Voss, K., Stephens, J., van der Harst, P., Chambers, J.C., Soranzo, N., Ouwehand, W.H., and Deloukas, P. (2013). Maps of open chromatin highlight cell type-restricted patterns of regulatory sequence variation at hematological trait loci. Genome research 23, 1130-1141.
4. Cowper-Sal lari, R., Zhang, X., Wright, J.B., Bailey, S.D., Cole, M.D., Eeckhoute, J., Moore, J.H., and Lupien, M. (2012). Breast cancer risk-associated SNPs modulate the affinity of chromatin for FOXA1 and alter gene expression. Nature genetics 44, 1191-1198.
5. Liu, J.Z., Almarri, M.A., Gaffney, D.J., Mells, G.F., Jostins, L., Cordell, H.J., Ducker, S.J., Day, D.B., Heneghan, M.A., Neuberger, J.M., et al. (2012). Dense fine-mapping study identifies new susceptibility loci for primary biliary cirrhosis. Nature genetics 44, 1137-1141.
6. Gerasimova, A., Chavez, L., Li, B., Seumois, G., Greenbaum, J., Rao, A., Vijayanand, P., and Peters, B. (2013). Predicting cell types and genetic variations contributing to disease by combining GWAS and epigenetic data. PloS one 8, e54359.
7. Maurano, M.T., Humbert, R., Rynes, E., Thurman, R.E., Haugen, E., Wang, H., Reynolds, A.P., Sandstrom, R., Qu, H., Brody, J., et al. (2012). Systematic localization of common disease-associated variation in regulatory DNA. Science 337, 1190-1195.
8. Lee, Y., Gamazon, E.R., Rebman, E., Lee, Y., Lee, S., Dolan, M.E., Cox, N.J., and Lussier, Y.A. (2012). Variants affecting exon skipping contribute to complex traits. PLoS genetics 8, e1002998.
9. Westra, H.J., Peters, M.J., Esko, T., Yaghootkar, H., Schurmann, C., Kettunen, J., Christiansen, M.W., Fairfax, B.P., Schramm, K., Powell, J.E., et al. (2013). Systematic identification of trans eQTLs as putative drivers of known disease associations. Nature genetics.
10. Dunham, I., Kundaje, A., Aldred, S.F., Collins, P.J., Davis, C.A., Doyle, F., Epstein, C.B., Frietze, S., Harrow, J., Kaul, R., et al. (2012). An integrated encyclopedia of DNA elements in the human genome. Nature 489, 57-74.
11. Anttila, V., Winsvold, B.S., Gormley, P., Kurth, T., Bettella, F., McMahon, G., Kallela, M., Malik, R., de Vries, B., Terwindt, G., et al. (2013). Genome-wide meta-analysis identifies new susceptibility loci for migraine. Nature genetics 45, 912-917.
12. Willer, C.J., Schmidt, E.M., Sengupta, S., Peloso, G.M., Gustafsson, S., Kanoni, S., Ganna, A., Chen, J., Buchkovich, M.L., Mora, S., et al. (2013). Discovery and refinement of loci associated with lipid levels. Nature genetics 45, 1274-1283.
13. Lappalainen, T., Sammeth, M., Friedlander, M.R., t Hoen, P.A., Monlong, J., Rivas, M.A., Gonzalez-Porta, M., Kurbatova, N., Griebel, T., Ferreira, P.G., et al. (2013). Transcriptome and genome sequencing uncovers functional variation in humans. Nature 501, 506-511.

14. Akhtar-Zaidi, B., Cowper-Sal-lari, R., Corradin, O., Saiakhova, A., Bartels, C.F., Balasubramanian, D., Myeroff, L., Lutterbaugh, J., Jarrar, A., Kalady, M.F., et al. (2012). Epigenomic enhancer profiling defines a signature of colon cancer. Science 336, 736-739.
15. Corradin, O., Saiakhova, A., Akhtar-Zaidi, B., Myeroff, L., Willis, J., Cowper-Sal lari, R., Lupien, M., Markowitz, S., and Scacheri, P.C. (2014). Combinatorial effects of multiple enhancer variants in linkage disequilibrium dictate levels of gene expression to confer susceptibility to common traits. Genome research 24, 1-13.
16. Pasquali, L., Gaulton, K.J., Rodriguez-Segui, S.A., Mularoni, L., Miguel-Escalada, I., Akerman, I., Tena, J.J., Moran, I., Gomez-Marin, C., van de Bunt, M., et al. (2014). Pancreatic islet enhancer clusters enriched in type 2 diabetes risk-associated variants. Nature genetics 46, 136-143.
17. Ernst, J., Kheradpour, P., Mikkelsen, T.S., Shoresh, N., Ward, L.D., Epstein, C.B., Zhang, X., Wang, L., Issner, R., Coyne, M., et al. (2011). Mapping and analysis of chromatin state dynamics in nine human cell types. Nature 473, 43-49.
18. Khurana, E., Fu, Y., Colonna, V., Mu, X.J., Kang, H.M., Lappalainen, T., Sboner, A., Lochovsky, L., Chen, J., Harmanci, A., et al. (2013). Integrative annotation of variants from 1092 humans: application to cancer genomics. Science 342, 1235587.
