# Supplementary material for: Disentangling the Effects of Colocalizing Genomic Annotations to Functionally Prioritize Non-coding Variants within Complex-Trait Loci
Source: Am J Hum Genet. 2015 Jul 2;97(1):139–52. doi: 10.1016/j.ajhg.2015.05.016 (PMC4572568; doi:10.1016/j.ajhg.2015.05.016)
Supplement: Document S2. Article plus Supplemental Data [file mmc3.pdf]

Disentangling the Effects of Colocalizing Genomic Annotations to Functionally Prioritize Non-coding Variants within Complex-Trait Loci

Gosia Trynka,[1,2,3,4] Harm-Jan Westra,[1,2,3] Kamil Slowikowski,[1,2,3,5] Xinli Hu,[1,2,3,6] Han Xu,[7] Barbara E. Stranger,[8,9] Robert J. Klein,[10] Buhm Han,[1,2,3,11,12] and Soumya Raychaudhuri[1,2,3,13,*]

Identifying genomic annotations that differentiate causal from trait-associated variants is essential to fine mapping disease loci. Although many studies have identified non-coding functional annotations that overlap disease-associated variants, these annotations often colocalize, complicating the ability to use these annotations for fine mapping causal variation. We developed a statistical approach (Genomic Annotation Shifter [GoShifter]) to assess whether enriched annotations are able to prioritize causal variation. GoShifter defines the null distribution of an annotation overlapping an allele by locally shifting annotations; this approach is less sensitive to biases arising from local genomic structure than commonly used enrichment methods that depend on SNP matching. Local shifting also allows GoShifter to identify independent causal effects from colocalizing annotations. Using GoShifter, we confirmed that variants in expression quantitative trail loci drive gene-expression changes though DNase-I hypersensitive sites (DHSs) near transcription start sites and independently through 3′ UTR regulation. We also showed that (1) 15%–36% of trait-associated loci map to DHSs independently of other annotations; (2) loci associated with breast cancer and rheumatoid arthritis harbor potentially causal variants near the summits of histone marks rather than full peak bodies; (3) variants associated with height are highly enriched in embryonic stem cell DHSs; and (4) we can effectively prioritize causal variation at specific loci.

Introduction

Functional annotations provide valuable information for prioritizing potential causal variants within complex-trait loci identified through genome-wide association studies (GWASs).[1–12] Profiles of such functional genomic annotations, including transcription factor binding sites and open chromatin regions from hundreds of cell types, are rapidly becoming available.[13–15] But, the most informative annotation is not always known. The most informative genomic annotations for fine mapping a particular trait are most likely related to mechanisms important for that trait. For example, binding sites for transcription factors that regulate key pathogenic pathways might prioritize variants for diseases,[5,7] and promoters active in a specific cell type might be able to prioritize expression quantitative trait locus (eQTL) variants from that cell type. Informative annotations such as these can be used for identifying likely causal variants, and these variants can then in turn be functionally interrogated for elucidating mechanisms underlying the trait.

Identifying the most informative annotations requires a robust statistical strategy that controls for two important types of potentially confounding genomic features: (1) local structure of genetic variation near SNP associations and (2) colocalization of multiple functional genomic annotations. First, trait-associated SNPs often map to regions with greater gene density, genetic variation, and linkage disequilibrium (LD) than the rest of the genome. Second, functional annotations that colocalize are often enriched within trait-associated loci. For example, DNase-I hypersensitive sites (DHSs) colocalize with exons,[16,17] and regulatory elements cluster together near and within gene transcripts. Therefore, an observed enrichment of one annotation might be the consequence of unaccounted colocalization with other annotation, thus confounding inferences of causality.

We developed Genomic Annotation Shifter (GoShifter), an enrichment test that controls for local genomic structure. GoShifter employs an intuitive method that locally shifts sites of tested features within each locus to generate a null distribution of annotations overlapping associated variants by chance. Other methods, such as Genome Structure Correction (GSC), assess the relationships between genomic features by shifting them.[1,18–20] Although GSC can assess the significance of overlap between two genomic features, it does not provide a clear application to individual GWAS loci and their local LD structure. Here, we apply the shifting approach to identify informative annotations for fine mapping GWAS

[1]Divisions of Genetics and Rheumatology, Department of Medicine, Brigham and Women's Hospital and Harvard Medical School, Boston, MA 02446, USA; [2]Partners Center for Personalized Genetic Medicine, Boston, MA 02446, USA; [3]Program in Medical and Population Genetics, Broad Institute of MIT and Harvard, Cambridge, MA 02142, USA; [4]Wellcome Trust Sanger Institute, Wellcome Trust Genome Campus, Cambridge CB10 1SA, UK; [5]Bioinformatics and Integrative Genomics, Harvard University, Cambridge, MA 02138, USA; [6]Harvard-MIT Division of Health Sciences and Technology, Boston, MA 02115, USA; [7]Department of Biostatistics and Computational Biology, Dana-Farber Cancer Institute and Harvard School of Public Health, Boston, MA 02215, USA; [8]Section of Genetic Medicine, Department of Medicine, University of Chicago, Chicago, IL 60637, USA; [9]Institute for Genomics and Systems Biology, University of Chicago, Chicago, IL 60637, USA; [10]Department of Genetics and Genomic Sciences, Icahn School of Medicine at Mount Sinai, New York, NY 10029, USA; [11]Asan Institute for Life Sciences, Asan Medical Center, Seoul 138-736, Republic of Korea; [12]Department of Medicine, University of Ulsan College of Medicine, Seoul 138-736, Republic of Korea; [13]Institute of Inflammation and Repair, University of Manchester, Manchester M13 9PT, UK
*Correspondence: soumya@broadinstitute.org
http://dx.doi.org/10.1016/j.ajhg.2015.05.016. ©2015 The Authors
This is an open access article under the CC BY license (http://creativecommons.org/licenses/by/4.0/).

loci. We benchmark the performance of GoShifter against that of commonly employed matching-based methods. These methods rely on inferring the enrichment of the SNP-annotation overlap in the observed data by contrasting it with the overlap in the null set of SNPs derived by random sampling of variants from the genome. In order to control for plausible genomic confounders, these methods sample SNPs by matching for a selection of defined genomic parameters. The selection of these parameters is based on the assumptions about possible analytical confounders. In contrast, GoShifter does not require prior knowledge because the null distribution is derived within the tested loci, ensuring that the density of SNPs, annotations, and the spatial distribution of genomic features are preserved.

We show that compared with commonly used SNP-matching-based methods, GoShifter is able to robustly identify informative annotations under a range of different scenarios. We show that matching-based approaches are prone to inflating observed enrichment values: we highlight that the lack of matching on SNPs in LD can lead to misleading results. Furthermore, we implemented a stratified test to distinguish contributions from two colocalized annotations. Using the local-shifting approach, GoShifter allows prioritization of loci by determining the most informative functional variants driving the observed enrichment.

Material and Methods

Assessing the Significance of Enrichment

We used three methods to assess the significance of enrichment: (1) local annotation shifting, (2) stratified local shifting, where we accounted for colocalization of a secondary annotation, and (3) SNP matching. We implemented (1) and (2) in GoShifter.

Local Annotation Shifting

To assess the statistical significance of an overlap between trait-associated SNPs and a genomic annotation X, we first identified all variants in LD with each index SNP ($r^2 > 0.8$ in 1000 Genomes Project European [EUR] samples[21]; Figure 1A) and determined the median size (in base pairs) of the tested annotation. We defined a locus as the region between the furthest linked SNPs and extended this region by twice the median size of the tested annotation. This ensured a sufficient size for testing the significance of an overlap within a locus defined by an index variant with no other variants in linkage. Next, we quantified the proportion of loci in which at least one SNP in LD overlapped X. We then randomly shifted X sites within each locus and quantified the proportion of loci overlapping X while fixing the locations of the SNPs (Figure 1B). We generated a null distribution from these proportions by repeating the shifting process over a large number of iterations. In each iteration, the magnitude of the shift was defined as a random integer sampled from a uniform distribution between 0 and the size of the locus in base pairs. We retained shifted annotations within the locus boundaries by "circularizing" the locus (i.e., as an annotation was shifted beyond the boundaries of a segment, it re-emerged on the other side of the segment). Circularization preserves the density of annotations and their spatial relationship within the tested region. We computed the p value as the proportion of iterations for which the number of overlapping loci was equal to or greater than that for the tested SNPs.

Stratified Enrichment of an Annotation

The stratified enrichment analysis assesses the significance of overlap of an annotation X while controlling for any overlap with a potentially colocalizing annotation Y (see Figure 1). We used a three-step approach: first, we fragmented each locus on the basis of the presence of Y while fixing the relative positions of the SNPs and annotation X. An X annotation site was split if it partially overlapped Y. Second, we concatenated these fragments, which yielded two distinct segments: (1) Y, which consisted of concatenated fragments of annotation Y, and (2) $\overline{Y}$, which lacked annotation Y (Figure 1C). This preserved the relationships and relative positions among X, Y, and the SNPs in the locus in both segments. Third, to generate the stratified null distribution for SNP overlap with X, we randomly shifted X within the Y and $\overline{Y}$ regions independently and quantified the proportion of loci that had at least one SNP that overlapped X in either region. To ensure that an annotation could not fall outside the segment boundaries, we circularized each of the Y and $\overline{Y}$ segments. As in the unstratified test, we defined the p value of the enrichment as the proportion of iterations where the number of loci with SNPs overlapping X exceeded the number of loci overlapping X prior to shifting. We note that, just as any form of stratified statistical analysis, the spatial restrictions on shifting X in a stratified manner might reduce power.

SNP Matching

Enrichment can also be determined through SNP matching, although a variety of parameters are used for matching SNPs in practice (Table S1). To compare the overlap with the null in a set of SNPs being tested for enrichment, we matched genomic variants on gene overlap (GEN), minor allele frequency (MAF; with 5% MAF bins), TSS proximity (bins defined by 500-bp, 2-kb, 5-kb, 10-kb, 20-kb, and 100-kb distances from the nearest TSS), transcription end site (TES) proximity (bins defined by 1-kb, 2-kb, 5-kb, 10-kb, 20-kb, and 100-kb distances from the TES of the same gene with the nearest TSS), and the number of SNPs in LD. If fewer than 20 SNPs were present in the sampling bin with matched SNPs, we expanded to the nearest LD bins while matching on the other properties. The arbitrary choice of 20 ensured that we had sufficient numbers of SNPs to sample for each SNP and relative independence between sample SNP sets. We constructed the null distribution by repeatedly quantifying the overlap between the annotation and the matched SNP sets. Then, we calculated the enrichment p value as the proportion of matched SNP sets with a number of overlaps equal to or greater than that of the tested SNPs. Because different genotyping platforms pose distinct biases because of their designs (efficiency of tagging, allele frequency of included SNPs, number of SNPs, and physical distribution in the genome), we derived the null distribution by sampling variants from a widely used commercial array (Illumina Omni 2.5).

Quantifying Observed Enrichment by Using Delta-Overlap

To quantify the effect size of the observed enrichment, we calculated the "delta-overlap" parameter: the difference between the observed proportion of loci overlapping an annotation and the mean of the proportion of loci overlapping the annotation under the null derived by local shifting. If there is no enrichment, the observed overlap will be close to the mean overlap under the

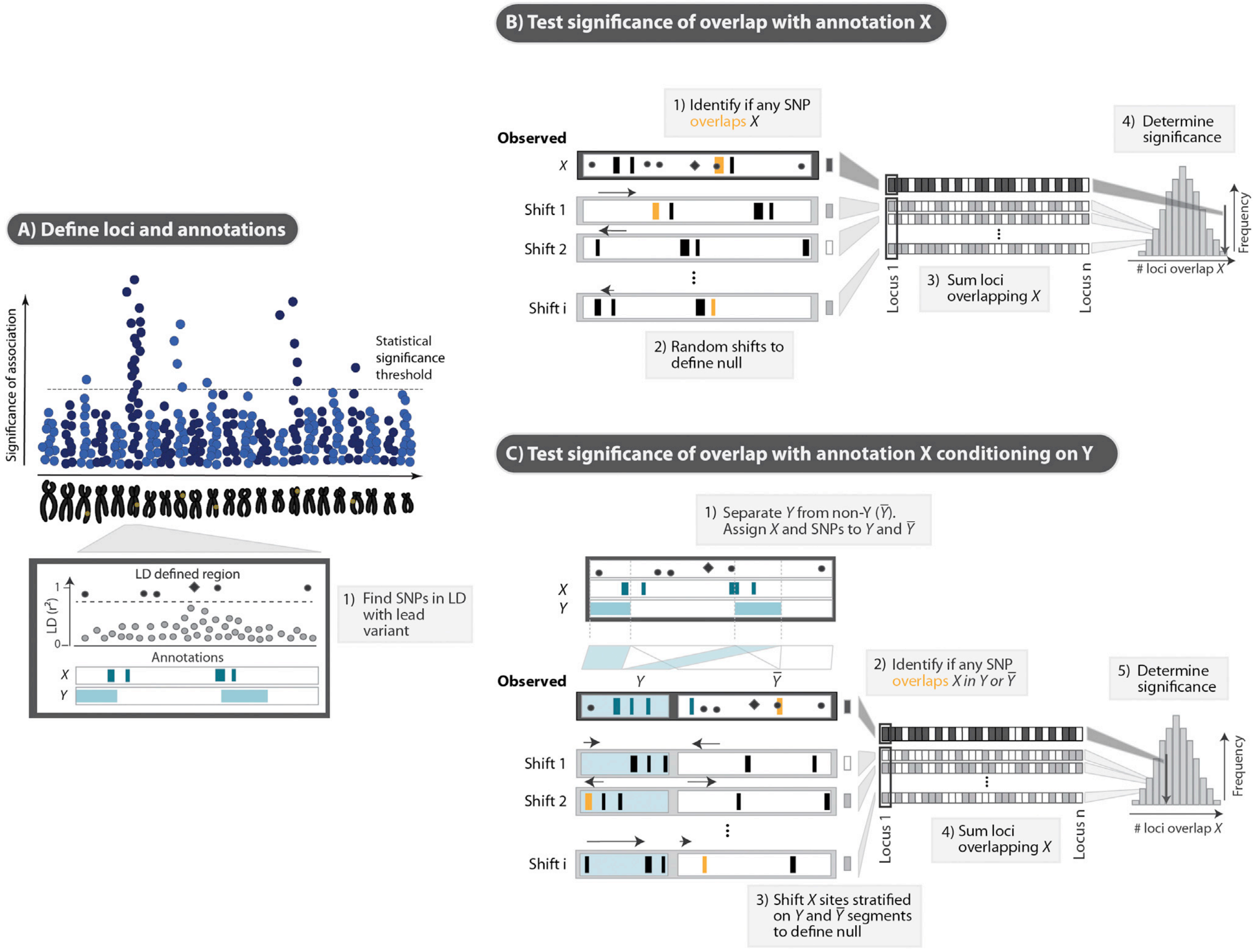

Figure 1. Schematic of the GoShifter Method

(A) To assess the statistical significance of an overlap between trait-associated SNPs and an annotation X, we start by using 1000 Genomes Project data to identify variants in LD ($r^2 > 0.8$) with each index SNP.

(B) We quantify the observed overlap: the proportion of loci where at least one linked SNP overlaps annotation X (shaded boxes). We estimate the significance of the observed overlap by comparing to a null distribution generated by random shifting of X sites (black arrows) within each locus. After each shift, we calculate the proportion of loci overlapping the annotation. To ensure that the same number of shifted annotations remains within locus boundaries, we circularize each region.

(C) To determine the significance of an overlap with annotation X independent of a possibly colocalizing annotation Y, we partition each locus into two types of fragments: those regions mapped by Y sites (light blue blocks) and those that lack them (denoted as $\overline{Y}$; white blocks). We join the respective Y and $\overline{Y}$ fragments into two independent continuous segments. To generate the null distribution, we shift annotation X separately within each of the two segments. For each iteration, we count the proportion of loci where any of the linked SNPs overlaps annotation X in either Y or $\overline{Y}$ segments to determine the significance of the observed overlap.

null, and delta-overlap will be close to 0. Conversely, larger delta-overlap values correspond to stronger enrichment. In practice, delta-overlap is independent of the number of SNPs in LD and the TSS or TES proximities of associated SNP sets (Figure S1).

Prioritizing Informative Loci by Using the Overlap Score

In order to identify individual loci where the overlap between a SNP and an annotation was particularly informative, we calculated an "overlap score" for each locus. The overlap score is the probability that each locus overlaps an annotation by chance. It is computed only for the loci that overlap the annotation in the observed data. Loci with low scores drive significant enrichment observations and are higher-priority candidates for further functional investigations. We defined the overlap score as l_s/n, where l_s is the number of shifting iterations for which at least one SNP within an individual locus overlaps the annotation, and n is the total number of iterations.

Genomic Annotations

Our study utilized DHS data, histone-modification data, and gene-annotation data compiled from publicly available resources.

DHSs

We used the DHS data from 80 experiments from ENCODE[13] and 137 experiments from the NIH Roadmap Epigenomics Project[14] (Table S2). We downloaded chromatin immunoprecipitation sequencing reads mapped to hg19 (UCSC Genome Browser) and merged reads from replicate samples. Using a corresponding input DNA library as the control if available, we ran MACS v.2.0[22] with default settings (false-discovery rate [FDR] = 0.01;

bandwidth = 300 bp) to identify significant peaks. In total, analysis of 217 experiments yielded 1,331,772 distinct autosomal DHSs, collectively spanning 16.4% of the genome. We combined DHS tracks across all cell types into a single consolidated DHS track by identifying the genomic positions that overlap DHSs in any cell type.

Histone Modifications

Similarly, we used MACS v.2.0 to call H3K4me3, H3K4me1, and H3K9ac peaks in 118, 114, and 50 tissues and cell-type samples, respectively, from the NIH Roadmap Epigenome[14] (see Table S2 for a detailed list of included tissues and cell types). If multiple replicates of the same tissue (input and control) were generated by the same center, we used these multiple BED files as input for MACS. For each experiment, we located the start and end of the peaks, as well as the summit regions (defined as ±100 bp around summits) called by MACS.

Gene Annotations

We defined gene annotations, including genes (whole transcripts including UTRs), exons, 5′ UTRs, 3′ UTRs, introns, and promoter regions, on the basis of RefSeq gene coordinates from the UCSC Genome Browser. We retained genes with at least one exon and at least one PubMed reference[23] to exclude poorly studied genes, pseudogenes, and falsely identified genes. In the resulting set of 18,183 genes, we identified exons, introns, and UTRs. We defined promoter regions as the first 500 bp upstream of the TSS. We note that DHS coverage for different gene features varied: 26% for exons, 73% for promoters, 75% for 5′ UTRs, 22% for 3′ UTRs, and 20% for introns.

Simulations

To assess GoShifter's sensitivity and specificity, we (1) defined sets of SNPs within functional regions, (2) generated sets of variants tagging these SNPs, as is common in GWASs, and (3) generated sets of SNPs with variable proportions of functional variants.

Defining Functional SNP Classes

Using a total of 6,830,225 common autosomal SNPs (MAF > 5% in Europeans in the 1000 Genomes Project), we grouped SNPs into seven functional categories: nonsynonymous, intronic, 3′ UTR, 5′ UTR, promoter (<500 bp from the TSS), intergenic (>5 kb from the TSS), and those residing within DHSs. To identify nonsynonymous variants, we used SIFT.[24]

Simulating SNP Sets Tagging Defined Functional GWAS Variants

We simulated 1,000 sets of 1,416 SNPs (to match the NHGRI GWAS Catalog SNP list), selected to overlap each of the predefined seven functional categories. In each set, variants were randomly selected from a given functional category. In order to mimic a typical GWAS approach, we then identified a tagging SNP that was in LD and was available on a commercial genotyping array (Illumina Human Omni2.5) for each functional variant. In total, 5,569,657 of the available SNPs were tagged ($r^2 \geq 0.5$) by 1,218,618 common (MAF ≥ 5%) variants on the Illumina Human Omni 2.5 array. If multiple SNPs were in LD with the functional variant, we selected the best tag with the greatest r^2. Finally, we required SNPs in the final set to be more than 100 kb apart from each other to ensure independence. As an alternative to simulating a sequencing-based study, we also simulated 200 sets of 1,416 causal SNPs for each of the predefined functional categories by selecting tagging SNPs with strong LD ($r^2 \geq 0.8$) from the EUR subset of the 1000 Genomes Project data. If multiple SNPs were in LD with the functional variant, we selected the best tag with the greatest r^2, and we selected the functional SNP itself when no tag SNP could be selected according to these criteria.

SNP Sets with Variable Proportions of Causal Functional Variants within DHSs

In addition to defining SNP sets derived from causal variants within a single annotation, we also defined SNP sets where causal variants were derived from two separate functional annotations. In these instances, we selected a proportion of causal variants from one annotation and selected the remainder from a second annotation.

Associated Variants

Disease-Associated Variants from the NHGRI GWAS Catalog

We obtained trait-associated SNPs from the GWAS Catalog[25] on November 5, 2013. We included only high-frequency (MAF > 5%) bi-allelic autosomal SNPs with a genome-wide significant ($p < 5 \times 10^{-8}$) association with any trait. We included only studies where Europeans contributed to the majority of the final samples to simplify LD calculations. We conducted LD calculations across the selected SNPs by using 379 EUR samples from the 1000 Genomes Project[21] and only bi-allelic SNPs with at least five copies of the minor allele. We ensured that these SNPs were independent by randomly excluding one SNP for each pair of SNPs if $r^2 > 0.1$ or if the distance between the SNPs was <100 kb. Finally, we excluded phenotypes with fewer than ten independent SNP associations after our filtering criteria. This resulted in 1,416 SNPs in our test set.

Variants Associated with Height, Rheumatoid Arthritis, and Breast Cancer

We used 689 SNPs associated with height,[26] 89 SNPs associated with rheumatoid arthritis (RA [MIM: 180300]) in Europeans alone or shared between Europeans and Japanese,[27] and 69 SNPs associated with breast cancer (MIM: 114480).[28]

eQTLs

We assembled a set of 923,022 *cis*-eQTL SNPs associated with whole-blood gene expression at a FDR of 0.5.[29] For each reported eQTL gene, we selected the single SNP most significantly associated with its expression and then performed LD pruning[30] (by using a window of 1,000 SNPs, sliding by one SNP at a time, and excluding one SNP in a pair if $r^2 > 0.1$) to ensure independence of the SNPs in our final SNP set. This resulted in a final dataset of 6,381 eQTL SNPs.

Results

GoShifter Is a Robust Method for Enrichment Testing

To evaluate GoShifter's performance in prioritizing annotations that identify causal variants, we would ideally use a set of trait-associated loci in which the causal variants and relevant driving genomic annotations are known. However, causal variants are known for only a handful of complex-trait loci. Therefore, to capture a wide range of possible models of causal variation, we simulated 1,000 sets of 1,416 SNPs by tagging functional SNPs selected from seven distinct functional genomic annotations: DHSs, promoters, 5′ UTRs, nonsynonymous SNPs in exons, 3′ UTRs, introns, and intergenic regions (Figure S2). We then tested these SNP sets for enrichment in DHSs. Pre-defining functional SNPs on the basis of specific driving annotations allowed us to assess the ability of a method to identify true enrichment and reject spurious overlap (Figure S3). An appropriate strategy should detect high DHS enrichment in sets designed to

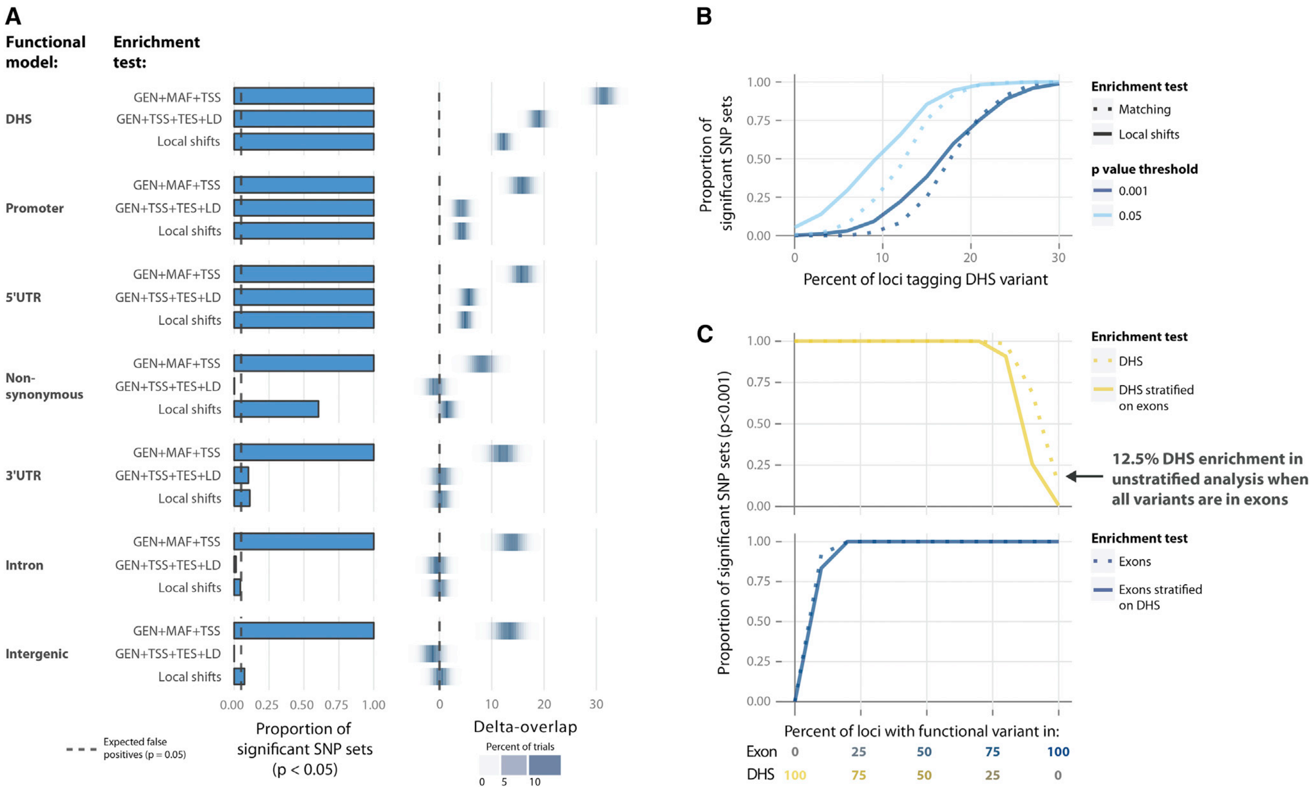

Figure 2. Comparison of Statistics between GoShifter and Matching-Based Tests
(A) We compared the performance of GoShifter with that of matching-based tests by using different parameters—(1) GEN, MAF, and TSS proximity and (2) GEN, LD, TSS proximity, and TES proximity—to match SNPs on. We generated sets of 1,416 SNPs tagging SNPs overlapping different genomic annotations; some SNP sets tagging SNPs in specific annotations (e.g., DHSs, promoter regions, 5′ UTRs, and nonsynonymous variants in exons) were enriched in DHSs, whereas others (e.g., 3′ UTRs, introns, and intergenic regions) were depleted in DHSs. For each functional model, we generated 1,000 sets of SNPs that we subsequently tested for enrichment in DHSs (left). The number of expected false positives at $p < 0.05$ is indicated by the dotted line. On the right, we plot the delta-overlap, which is the difference between the proportions of SNPs overlapping an annotation in the actual data and the proportion of SNPs overlapping an annotation in the null distribution.
(B) We generated sets of 1,416 SNPs with variable proportions of variants within DHSs (increments of 5% and 1,000 sets per increment). We compared the power to detect significant enrichment in DHSs for each increment (i.e., the proportion of significant SNP sets) between GoShifter and the best-performing matching-based strategy (GEN, LD, TSS proximity, and TES proximity) for two significance levels ($p < 0.05$ and $p < 0.001$).
(C) To test the performance of GoShifter, we generated sets of 1,416 SNPs with varying proportions of variants in either exons or DHSs (with increments of 5% in either annotation and 1,000 sets per increment). We then used GoShifter to analyze the enrichment (at $p < 0.001$) in DHSs stratified on exons (upper panel) and vice versa (lower panel).

tag functional variants in regulatory regions (DHSs, promoters, and 5′ UTRs), modest enrichment in nonsynonymous variants in exons and 3′ UTRs (which colocalize with DHSs),[16,17] and no enrichment at introns or intergenic regions (Material and Methods).

We observed that GoShifter was well powered to detect significant enrichment in simulated SNP sets that tagged variants in DHSs, promoters, and 5′ UTRs: 100% of such SNP sets obtained $p < 0.001$ according to 1,000 shifting iterations (Figure 2A; Figure S4). By chance, we would expect 5% of the SNP sets tagging variants in intronic and intergenic regions to obtain $p < 0.05$; indeed, we observed that 4.44% and 7.4%, respectively, obtained $p < 0.05$ (Figure 2A; Table S3). The SNP sets tagging 3′ UTRs and nonsynonymous variants appropriately showed modest enrichment (60% and 11% of these SNP sets, respectively, obtained $p < 0.05$).

The analyses to benchmark GoShifter were based on simulated GWAS SNP sets ascertained from a commercial array. To assess whether ascertaining tag SNPs from commercial genotyping arrays would affect observed statistics, we also evaluated GoShifter's performance by using sequencing SNPs (from the 1000 Genomes Project).[21] We observed no significant difference for GoShifter's performance under genotyping or sequencing scenarios (Figure S5). This indicates that GoShifter's performance is robust to the intrinsic biases in SNP ascertainment of commercial genotyping arrays.

We then compared the results of GoShifter to those of the more commonly used matching-based enrichment tests[2,5,7–10,12,13,19,29,31–38] (Table S1). These methods typically match SNPs on GEN, MAF, and TSS proximity.[2,7,12,13,19,32,35] We observed that when we matched SNPs on these parameters, all simulated SNP sets,

including 100% of those with functional variants derived from intergenic regions, obtained $p < 0.05$ for DHS enrichment (Figure 2A; Table S3). This suggests that selecting variants on GEN, MAF, and TSS proximity might be insufficient to control false-positive rates in the assessment of DHSs.

We then investigated whether matching on other combinations of SNP features was more effective. As expected, we observed that the results were highly sensitive to the choice of specific matching parameters. We noted that the number of SNPs in LD was critical for appropriate statistical performance (Figure S4). Although MAF was frequently included, it had little effect. We observed that when SNPs were derived from 3′ UTRs, matching on TES proximity substantially decreased the inflated statistics. Ultimately, we identified that matching on the combination of GEN, TSS proximity, TES proximity, and LD adequately controlled type I error if the SNPs were selected from non-regulatory regions (e.g., intergenic or intronic regions).

After determining the best-performing SNP-matching strategy, we wanted to more precisely quantify differences in sensitivity between GoShifter and matching-based tests. For this purpose, we generated SNP sets where only a minority of variants tagged functional variants within DHSs. We incremented the percentage of loci tagging functional variants in the simulated SNP set by 3%. These simulations represented a test of the methods for detecting enrichment under a range of more-challenging circumstances. For a set in which 10% of SNPs tagged DHSs, GoShifter had 55% power to detect enrichment, but only 31% power with stringent SNP matching (Figure 2B). For a set in which 10% of SNPs tagged DHSs, GoShifter had 55% power to detect enrichment, but only 31% power with stringent SNP matching (Figure 2B).

Stratified Analysis Distinguishes Effects of Colocalizing Annotations

To test the ability of GoShifter to control for the effects of colocalized annotations, we examined two scenarios.

First, we tested the enrichment of different annotations in 1,000 sets of 1,416 SNPs tagging exonic variants. Although we observed significant enrichment of exons across all SNP sets ($p < 0.05$), 60% also showed DHS enrichment (Figure 2A). This secondary enrichment was a result of colocalization between the DHSs and exons.[16,17] Testing for DHS enrichment after stratifying on exons resulted in a much lower type I error rate, such that 10.2% of SNP sets obtained $p < 0.05$. Similarly, at a more stringent significance level ($p < 0.001$), we observed DHS enrichment in 12.5% of the SNP sets and 0.7% after stratifying on exons (Figure 2C).

Second, we assessed 1,000 sets of 1,416 SNPs defined to tag variants within DHSs. DHSs tend to overlap regions mapped by H3K4me3 because they both colocalize with active promoter regions. When testing for enrichment, we would therefore expect to observe significant signal for both of these annotations. However, because we know that functional signals were drawn from DHSs, an adequate method should not detect significant enrichment in H3K4me3 when it is stratified on the lead signal from DHSs. Whereas all SNP sets were enriched in DHSs at $p < 0.05$, 98% of the SNP sets were also enriched in H3K4me3 sites (a histone mark known to highlight promoters and colocalize with DHSs). As expected, after we stratified on DHSs, only 1.3% of the SNP sets showed H3K4me3 enrichment at $p < 0.05$, confirming that H3K4me3 enrichment is entirely dependent on DHS colocalization. Conversely, when tested for DHS enrichment stratifying on H3K4me3, 100% of the SNP sets remained significant. This confirmed that signal in H3K4me3 was primarily explained by the DHS enrichment.

The stratified analysis modestly reduced power to detect enrichment effects: for SNP sets where functional variants were selected from both DHSs and exons, stratifying on exons reduced power to detect DHS enrichment at $p < 0.001$ only when $\leq$20% of the functional variants were from DHSs (Figure 2C). However, stratifying on DHSs did not limit the power to detect exon enrichment.

We also assessed whether SNP-matching approaches effectively account for colocalization of annotations. We again considered the sets of DHS-tagging SNPs and tested for H3K4me3 enrichment. To control for the DHS effect, we used the most appropriate matching strategy (GEN, TSS proximity, TES proximity, and LD) and included an extra matching parameter (DHS). We observed that 100% of SNP sets still obtained H3K4me3 enrichment at $p < 0.05$. We concluded that SNP-matching tests cannot easily control for colocalization of annotations (Figure S6).

GoShifter Effectively Annotates eQTL SNPs

As a proof of concept, we applied our approach to eQTL variants, given that eQTLs are known to localize close to TSSs and to be enriched in DHSs.[39–43]

First, we used GoShifter to assess the enrichment of eQTLs in DHSs at various distances to the TSS (Figure 3A), various histone marks (H3K9ac, H3K4me3, and H3K4me1) associated with active gene regulation, and genes (Figure 3B). We also included 3′ UTRs because they have been previously suggested to independently account for a proportion of eQTL signal.[29,39] We observed that each of these annotations was enriched in eQTLs ($p < 0.05$). To better localize the source of enrichment with respect to the TSS, we stratified the TSS-distance window for DHSs; we observed only significant results within 500 bp of the TSS ($p = 2 \times 10^{-4}$; Figure 3A), consistent with earlier studies. We then assessed the enrichment of the other annotations while stratifying for DHSs. We observed that the enrichment for genes and each of the histone marks, with the exception of H3K4me1 ($p = 0.02$) and 3′ UTRs ($p = 6 \times 10^{-3}$), became insignificant (Figure 3B). However, DHS enrichment remained significant after we stratified on each of the annotations, indicating that eQTLs are enriched in DHSs independently of the other annotations. This suggests that eQTLs might act through mechanisms

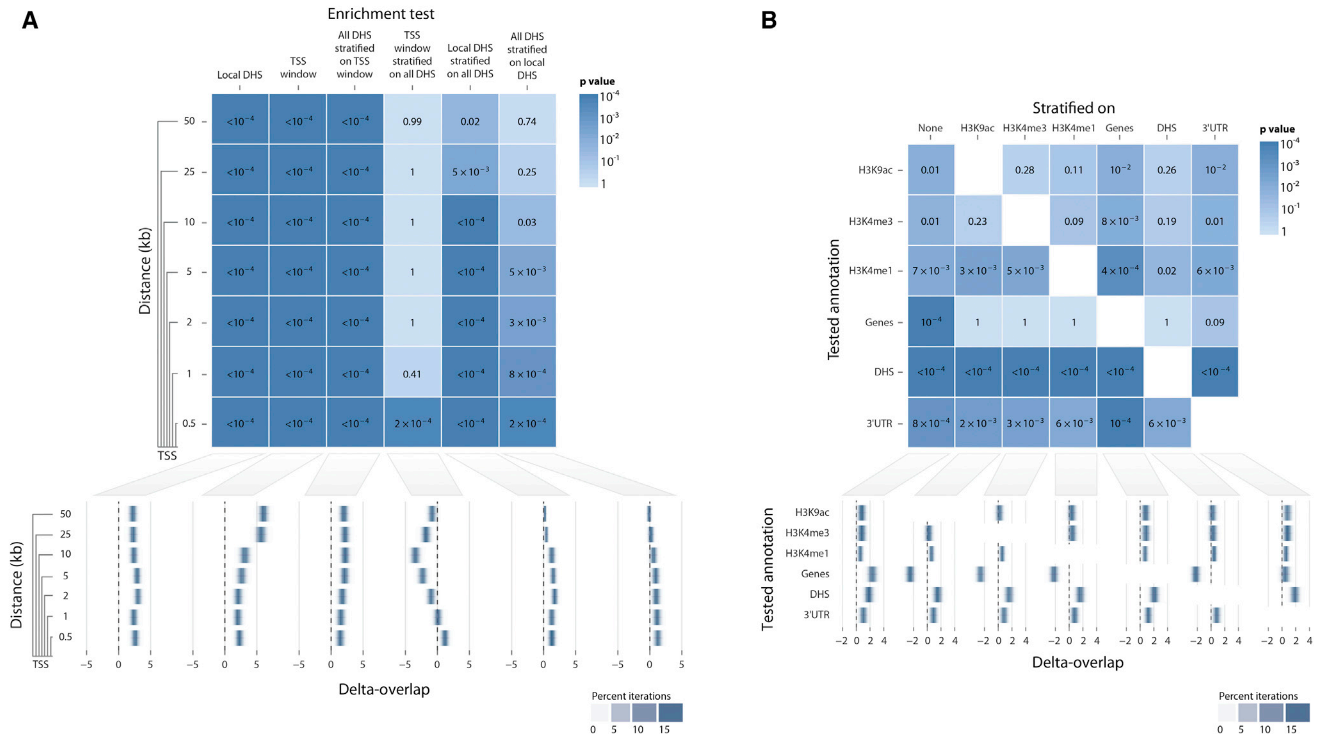

Figure 3. eQTL Variants Localize to DHSs near TSSs

(A) To test the performance of GoShifter on real data, we analyzed the enrichment of 6,380 eQTLs with local DHSs at various distances (varying between 0.5 and 50 kb) to the TSS by using 10,000 random shifts. The p values for each analysis are in the top panel, and the delta-overlap measures are in the bottom panel (a higher value denotes a higher proportion of significant loci than in the null distribution).

(B) We tested enrichment of these eQTLs in various other regulatory marks (H3K9ac, H3K4me3 H3K4me1, and DHSs) associated with active transcription (10,000 random shifts) and overlap with genes and 3′ UTRs. We tested each annotation in an unstratified analysis, and we also tested for enrichment stratifying on each of the other annotations. When we tested for gene-transcript enrichment by stratifying on regulatory annotations, negative delta-overlap values indicated that eQTL SNPs were primarily captured by the regulatory annotations and depleted in gene transcripts (except for 3′ UTRs).

independent of genetic variation in DHSs. For 3′ UTRs, these mechanisms might include alterations in miRNA binding sites.[44–46]

Quantifying the Proportion of GWAS Catalog SNPs with DHS Causal Variants

We assessed 1,416 independent SNP associations from the NHGRI GWAS Catalog[25] for overlap with different annotations (Material and Methods). We observed enrichment at DHSs ($p < 10^{-4}$), at genes, at H3K4me3 and H3K4me1 marks, and also at 5- and 10-kb windows around TSSs (Figure 4A). Pairwise stratified tests showed that DHSs were enriched independently of other annotations ($p \leq 7 \times 10^{-4}$). In contrast, the enrichment of gene transcripts and TSSs was not significant after stratification on DHSs. Both H3K4me1 and H3K4me3 retained nominal enrichment independently of each other and DHSs ($p \leq 0.05$; Figure 4B). These results suggest that causal disease-associated variants in DHSs and H3K4me3 and H3K4me1 marks function through three independent mechanisms.

We aimed to accurately determine the proportion of GWAS loci that tag variants in DHSs by using the GoShifter-derived delta-overlap parameter (Material and Methods), which quantifies the strength of observed enrichment (Figure S1). We calculated the delta-overlap for the GWAS Catalog SNPs and DHSs to be 3.17% (Figure 4C). We then sought to infer the proportion of loci that overlap DHSs from the observed delta-overlap value. We simulated 1,000 SNP sets (the same size as the GWAS Catalog set) with 0%–45% of causal variants in DHSs by using 3% increments and calculated their corresponding delta-overlap values. We selected simulated sets that had a delta-overlap within the range of that of the GWAS Catalog (delta-overlap ± 0.2) and created a probability distribution of the proportion of causal variants within DHSs. From this distribution, we estimated the mean and 95% confidence interval for the proportion of causal variants in DHSs for the GWAS Catalog. We determined that the delta-overlap of 3.17% corresponded to 15%–36% causal variants in DHSs (95% confidence; Figure 4D). Recent studies have estimated that around 80% of trait-associated variants (or the SNPs in LD) within the GWAS Catalog overlap DHSs[2,12,13]; our estimates suggest that DHS enrichment might be more modest than previously reported.

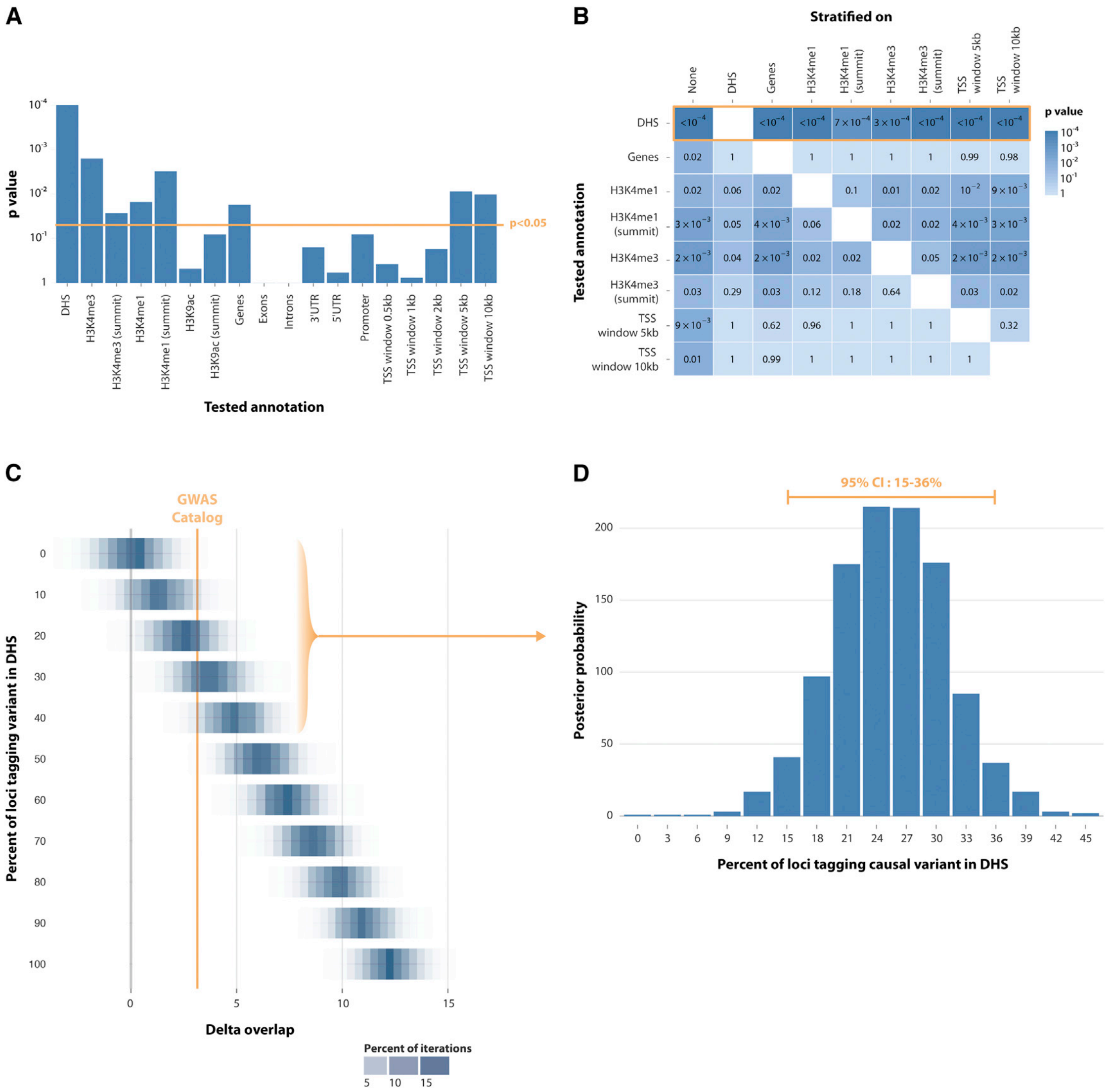

Figure 4. Quantifying the Proportion of Causal GWAS Catalog Variants Derived from DHSs

(A) We assessed the enrichment of 1,416 independent GWAS Catalog SNPs in various genomic annotations by using GoShifter with 10,000 local shifts. We observed strong enrichment of DHSs ($p < 10^{-4}$) and nominal enrichment ($p < 0.05$, yellow line) of H3K4me3, H3K4me1, genes, and distance to the TSS (5 and 10 kb).

(B) We performed pairwise stratified analysis for significantly enriched annotations. DHSs showed a strong residual enrichment ($p < 7 \times 10^{-4}$) after stratification on each of the other annotations.

(C) We generated sets of 1,416 SNPs overlapping an increasing proportion of DHSs (5% increments and 1,000 sets per increment) and determined the delta-overlap per set, yielding a delta-overlap distribution per DHS-overlap increment. We then determined the delta-overlap for the real GWAS Catalog to be 3.17 (dotted line), which corresponds to 15%–36% of loci with causal variants within DHSs (D) within the 95% confidence interval.

RA and Breast Cancer Associations Are Enriched at the Summits of Cell-Type-Specific Histone Marks

We examined two phenotypes to test GoShifter's ability to identify cell-type-specific functional variants. To ensure that GoShifter was powered to detect significant enrichments (Figure S7), we selected phenotypes with over 50 associated variants at the commonly applied genome-wide significance threshold ($p < 5 \times 10^{-8}$). We first tested 88 SNPs associated with RA[27] for enrichment of H3K4me3. We focused on $CD4^{+}$ memory T cells given recent observations of cell-type-specific gene expression and eQTLs within these loci.[1,47,48] We observed no enrichment ($p = 0.17$) when we used the peak bodies of H3K4me3 in either $CD4^{+}$ memory T cells ($p = 0.17$) or in an aggregate of all 118 cell types from our datasets ($p = 0.14$; Figure 5A). Because the median width of H3K4me3 peak bodies varied widely (110–86,490 bp), we examined the summit regions (±100 bp from the H3K4me3 summits), where active gene-regulatory elements are most likely located.[49] In the summit regions, we observed significant enrichment both in the 118 aggregate cell types ($p = 0.044$) and in $CD4^{+}$ memory T cells specifically ($p = 1.6 \times 10^{-3}$). The $CD4^{+}$ memory T cell signal remained significant after stratification on the summit regions of the other 117

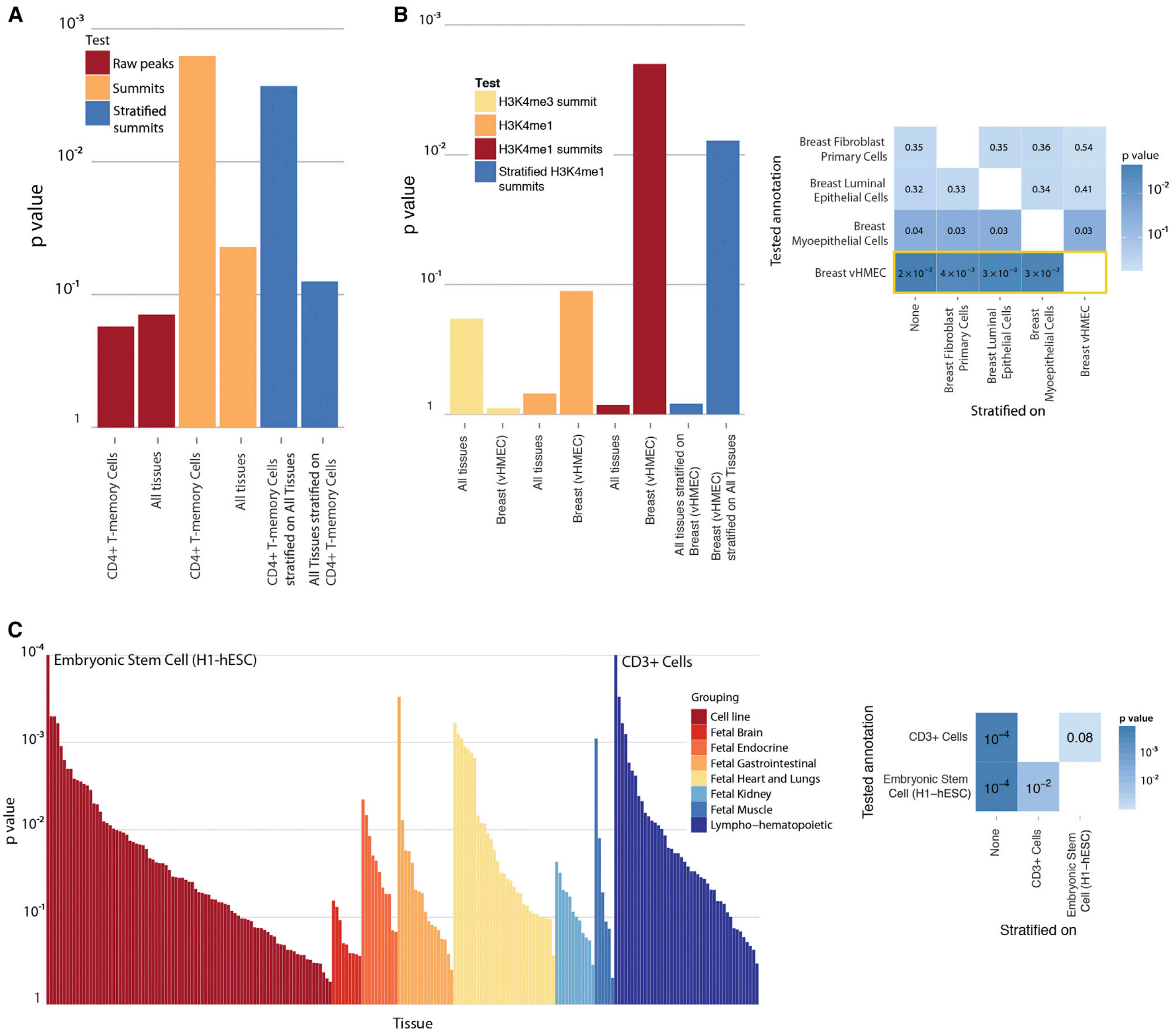

Figure 5. Enrichment Results for Three Selected Sets of Trait-Associated SNPs

(A) We examined the enrichment of 88 RA-associated variants with H3K4me3 in $CD4^{+}$ T memory cells and in an aggregate of 118 different cell types and tissues. We assessed raw peaks (peak bodies) and summit regions (± 100 bp from the summit). We observed a nominally significant enrichment in the aggregate of cell types and tissues ($p = 0.044$) and a pronounced enrichment within $CD4^{+}$ T memory cells ($p = 1.6 \times 10^{-3}$). Stratified analysis indicated that the enrichment signal was driven by $CD4^{+}$ T memory cells: the significance of the cell-type-aggregate enrichment decreased ($p = 0.08$) when we stratified on $CD4^{+}$ T cells, but not vice versa ($p = 2.7 \times 10^{-3}$).

(B) We assessed the enrichment of 69 breast-cancer-associated variants with various histone marks (H3K4me3 and H3K4me1) in the 118 tissues and cell types. Breast-cancer-associated SNPs were highly enriched ($p = 2 \times 10^{-3}$) in summit regions of H3K4me1 peaks in vHMECs (left panel), but not in other cells, H3K4me3 summit regions ($p > 0.4$), or H3K4me1 peak bodies. The stratified enrichment analysis indicated that the enrichment of H3K4me1 summit regions in vHMECs was independent of the H3K4me1 summit regions in the aggregated cell types and tissues. The H3K4me1 enrichment in vHMECs within summit regions was maintained when we stratified on summit regions from other breast tissues and cell types ($p < 3.6 \times 10^{-3}$; right panel).

(C) Similarly, we assessed enrichment of 697 SNPs associated with height in DHSs from 217 different tissues. The height-associated SNPs showed the highest enrichment of DHSs in embryonic stem cells ($p < 10^{-4}$) and $CD3^{+}$ cells ($p < 10^{-4}$) from cord blood (left panel). However, the $CD3^{+}$ cell DHS enrichment diminished after stratification on embryonic stem cells ($p = 0.08$), whereas embryonic stem cells retained significance after stratification on $CD3^{+}$ cells ($p = 9.6 \times 10^{-3}$; right panel).

cell types ($p = 2.7 \times 10^{-3}$). In contrast, the other cell types did not retain significant enrichment after stratification on $CD4^{+}$ memory T cells ($p = 0.08$). These results suggest that H3K4me3 summit regions in $CD4^{+}$ memory T cells could help prioritize causal variants in RA-associated loci.

Similarly, we examined 69 SNPs[28] associated with breast cancer for enrichment of H3K4me3. Summit regions were not enriched ($p > 0.4$) in any of the three breast tissues present in our dataset. We therefore tested for enrichment of another active regulatory mark,

H3K4me1, for which there were four breast tissues in our dataset. The peak bodies of this mark were nominally enriched ($p = 0.034$) only in breast myoepithelial cells. However, when we used H3K4me1 summit regions, we observed significant enrichment ($p = 2 \times 10^{-3}$) in variant human mammary epithelial cells (vHMECs; Figure 5B). We performed pairwise stratified enrichment tests across the four breast tissues and found that the vHMEC summit regions retained significance ($p < 3.6 \times 10^{-3}$) after stratification on peak summits from each of the other three breast tissues. We found that none of the DHS samples in our dataset showed nominally significant enrichment.

Stratified Analysis Can Indicate Relevant Cell Types for Height

We assessed 697 SNPs associated with adult human height,[26] a highly polygenic trait without clearly established relevant cell types. When we examined aggregated DHSs from 217 cell types collectively, we observed nominal evidence of overlap ($p = 0.019$). Individually, many tissues, including 13 at $p < 10^{-3}$, demonstrated some evidence of overlap (Figure 5C). We observed the strongest enrichment ($p < 10^{-4}$) in embryonic stem cells (H1-hESCs) and primary $CD3^{+}$ cells from cord blood. The enrichment in H1-hESCs remained significant after stratification on cord-blood $CD3^{+}$ cells ($p = 9.6 \times 10^{-3}$), but the converse enrichment ($p = 0.08$) did not. These results suggest that examining DHSs in embryonic stem cells or a related cell type might be informative for fine mapping height-associated loci for potential causal variants.

The Locus Overlap Score Can Be Used for Prioritizing Trait-Associated Loci

After the most significantly enriched annotations are identified, the loci contributing the most to such enrichment are those with the lowest overlap score (Figure S8). A locus that obtains a low overlap score would typically have only a few variants linked to the index SNP and sparse density of the annotation. These are the loci where variants might be most effectively prioritized.

For example, in breast cancer, the locus with the best (lowest) overlap score (0.097) was rs889312 (Figure 6). That SNP is in LD with seven other variants, of which only rs1862626 overlaps a vHMEC H3K4me1 summit region. This SNP is upstream of *MAP3K1* (MIM: 600982) and modifies a predicted binding site for estrogen receptor alpha (ER-α),[50] consistent with the well-established role of estrogen-mediated signaling in breast cancer progression.[51,52] For height, rs11677466 showed the best overlap score (0.026; Figure 6). This SNP itself overlaps an embryonic stem cell DHS that is a known binding site for HNF4α,[53–55] a transcription factor that plays important roles in metabolic regulation and stem cell differentiation. Functional follow-up will be necessary to further validate these variants. The specific annotations indicate the type of regulatory element driving these associations and define the cell type in which they are active.

Discussion

Here, we have presented GoShifter, a method that enables the identification of annotations that are the most informative in distinguishing causal variants from associated ones. GoShifter stringently controls for local features by shifting annotations within trait-associated loci. Our method represents an important advance over current approaches in its ability to assess independent effects from colocalizing annotations.

GoShifter is suited to investigate whether a specific annotation might be effective in fine mapping known genetic loci for a trait. Consequently, GoShifter does tend to favor high-resolution annotations (e.g., annotations with a small average size). But in certain instances, large annotations (e.g., super enhancers or large gene sets) might be important indicators that separate trait-associated variation from non-trait-associated variation.[56] Although informative, these annotations might not be particularly effective for fine mapping. Under these circumstances, SNP matching might be a necessity. We note with caution, however, that the importance of individual matching parameters might be different depending on which specific annotations are being tested. Importantly, matching on the number of SNPs in LD will be critical: the number of SNPs that a variant is in LD with will be proportional to the chance that one of those variants overlaps an annotation; consequently, failing to control for LD invariably yields inflated results. In certain instances, controlling for LD alone might substantially mitigate type I error, but in most instances, matching on LD alone will not be adequate. Additional parameters should be carefully considered and evaluated (e.g., by simulation experiments) when SNP-matching-based enrichment tests are used. For example, in the assessment of DHS enrichment, our results demonstrate that GEN, TSS proximity, and TES proximity are additional important parameters. We speculate that to accurately detect the enrichment in exons, it might be necessary to further match on other parameters, such as the number of exons and gene length.

In this study, we examined variants in LD ($r^2 > 0.8$) with an index SNP for overlap with an annotation. This is a widely used approach, but it has limitations. In many instances, index SNPs within loci that have been detected by sparse genotyping or in relatively smaller studies have a lower degree of linkage than $r^2 > 0.8$ to a causal variant. This is particularly true in complex genetic regions, where multiple haplotypes might be driving an association, such as within the major histocompatibility complex in autoimmune diseases[57] or *IRF5* (MIM: 607218) in systemic lupus erythematosus.[58] One approach to increasing the sensitivity of enrichment methods would be to weight annotation overlaps by the posterior probabilities that associated

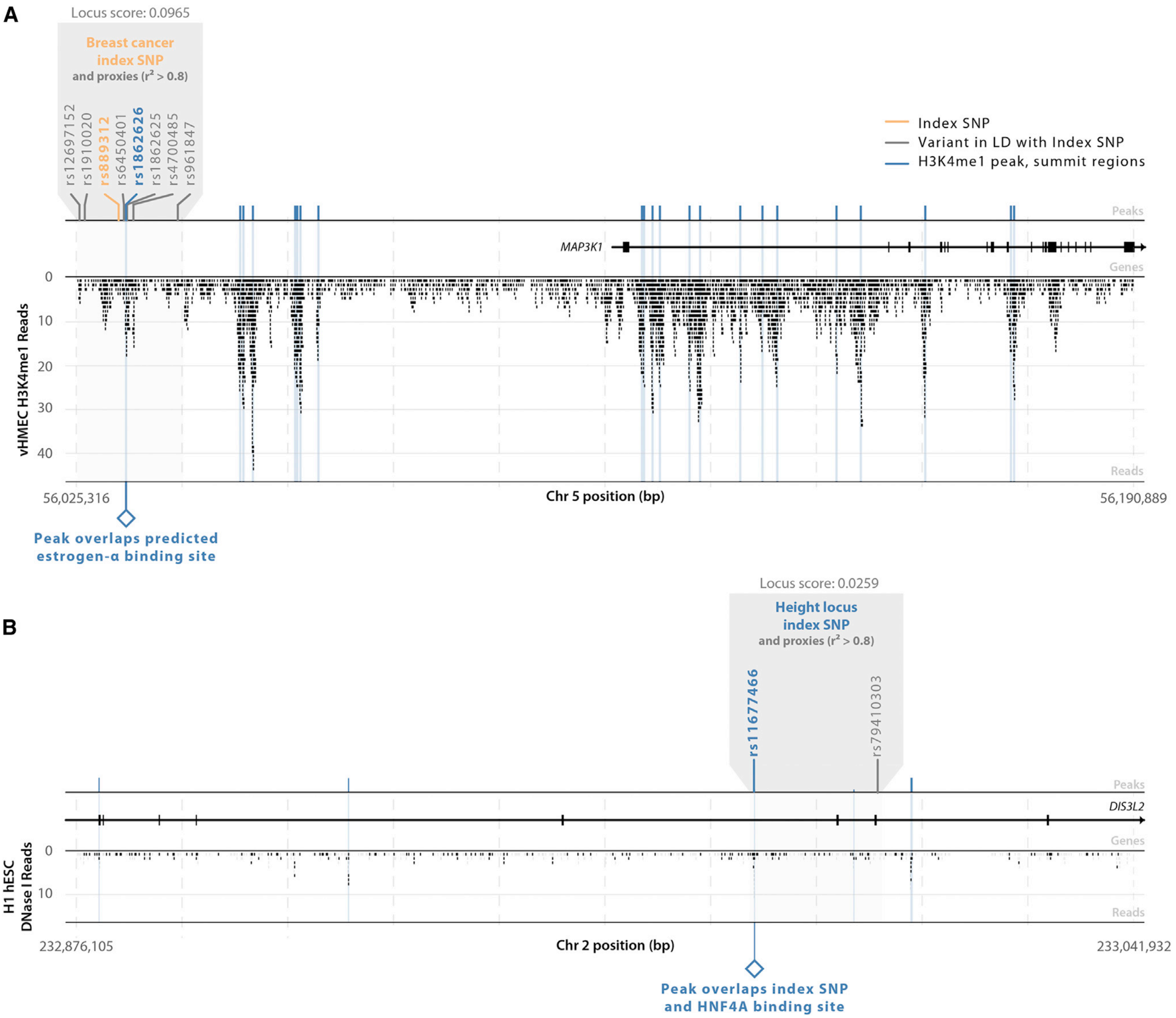

Figure 6. Locus Plots Showing the Peaks, Variants, and Reads in Two Trait-Associated Loci
(A) The SNP rs889312 defines the locus with the best overlap score among breast cancer SNP associations. This SNP is in LD ($r^2 > 0.8$) with a variant (rs1862626) that overlaps the summit region of an H3K4me1 peak. This peak overlaps a predicted ER-α binding site. The associated locus is located upstream of potential oncogene *MAP3K1*.
(B) Of the height-associated SNPs, rs11677466 defines the locus with the best overlap score and is located in an exon of *DIS3L2* (MIM: 614184). This SNP overlaps a DHS peak, which also overlaps a known HNF4α binding site.

SNPs are the individual causal SNPs.[59] GoShifter can be used again to make significance assessments by shifting annotations and reassessing weighted overlap to define a null distribution.

We present examples to illustrate how stratified analysis might help to determine whether fundamentally different functions (such as regulatory versus coding variation) might be driving complex-disease associations. In many instances, different annotations colocalize because they are derived from different molecular assays that query the same genomic functional process. For example, H3K4me1 marks and bidirectional transcription[60] are both signatures of enhancers. In these instances, stratified analysis can be used to determine assays or cell types with the most informative annotations. Indeed, it could even be the case that multiple assays are independently informative, and combining them could be the most powerful approach. Stratified analysis offers not only a clear strategy for evaluating individual annotations against each other but also for assessing whether combinations of annotations are more informative than any individual annotation.

Applying GoShifter, we observed that different annotations play dominant roles in different complex traits. The eQTL results have highlighted that in addition to gene-regulatory regions and protein-coding genes, other mechanisms might be mediating causal variation for complex phenotypes. The observed enrichment of 3′ UTRs in eQTLs

suggests that post-transcriptional gene regulation is one such mechanism by which genomic variation affects gene-transcript levels. Other functional genomic features, such as non-coding RNAs and copy-number variation, could also be driving complex phenotypic and disease variation.[39,44–46]

Our results indicate that SNPs represented in the GWAS Catalog are globally enriched within DHSs. However, this is not representative of enrichment for individual phenotypes and in fact might be driven by a selection of phenotypes with a large number of associated variants. When we applied GoShifter to variants associated with height, we observed strong DHS enrichment specifically in embryonic stem cells, whereas breast cancer variants were enriched in H3K4me1 but not DHSs. These could be partly due to the fact that different trait-relevant tissues were mapped with different assays. Nevertheless, variants associated with different phenotypes are likely to act through various mechanisms, resulting in differential enrichment of annotations. These results also show that our stratified approach can be applied to narrow down the specific cell type in which disease-associated variants function, even from within a single disease tissue of interest. The strong enrichment of breast-cancer- and RA-associated loci within the summit regions of histone marks is consistent with the well-described functional significance of the summit regions.[49]

We expect that the applicability of GoShifter will expand as relevant functional annotations increase over time. Once our approach identifies independently informative functional annotations by analyzing a large set of associated variants, a powerful aspect of GoShifter is the ability to identify the specific loci that drive the enrichment statistics. Combining the identification of the functional annotations associated with a trait and the identification of the loci that drive that association should facilitate effective and focused experimental interrogation of these loci.

Software is available as the GoShifter package, written in Python-2.7.

Supplemental Data

Supplemental Data include eight figures and three tables and can be found with this article online at http://dx.doi.org/10.1016/j.ajhg.2015.05.016.

Acknowledgments

We acknowledge the GIANT consortium for providing us access to the height SNPs. We also acknowledge Joel Hirschhorn, X. Shirley Liu, Mark Daly, Tonu Esko, Dorothee Diogo, and Joshua Randall for helpful scientific discussions. This work was supported by funding from the NIH (NIAMS-1R01AR063759 [S.R.], 1UH2AR067677-01 [S.R.], 1U19AI111224-01 [S.R.], and NHGRI-1U01HG0070033 [R.J.K.]), a Doris Duke Clinical Scientist Development Award (S.R.), the Rubicon grant from the Netherlands Organization for Scientific Research (G.T.), and the Wellcome Trust Sanger Institute (WT098051 [G.T.]).

Received: March 9, 2015
Accepted: May 26, 2015
Published: July 2, 2015

Web Resources

The URLs for data presented herein are as follows:

1000 Genomes, http://www.1000genomes.org/
GoShifter, http://www.broadinstitute.org/mpg/goshifter/
OMIM, http://www.omim.org/
UCSC Genome Browser, http://genome.ucsc.edu/

References

1. Trynka, G., Sandor, C., Han, B., Xu, H., Stranger, B.E., Liu, X.S., and Raychaudhuri, S. (2013). Chromatin marks identify critical cell types for fine mapping complex trait variants. Nat. Genet. *45*, 124–130.
2. Maurano, M.T., Humbert, R., Rynes, E., Thurman, R.E., Haugen, E., Wang, H., Reynolds, A.P., Sandstrom, R., Qu, H., Brody, J., et al. (2012). Systematic localization of common disease-associated variation in regulatory DNA. Science *337*, 1190–1195.
3. Adrianto, I., Wen, F., Templeton, A., Wiley, G., King, J.B., Lessard, C.J., Bates, J.S., Hu, Y., Kelly, J.A., Kaufman, K.M., et al.; BIOLUPUS and GENLES Networks (2011). Association of a functional variant downstream of TNFAIP3 with systemic lupus erythematosus. Nat. Genet. *43*, 253–258.
4. Musunuru, K., Strong, A., Frank-Kamenetsky, M., Lee, N.E., Ahfeldt, T., Sachs, K.V., Li, X., Li, H., Kuperwasser, N., Ruda, V.M., et al. (2010). From noncoding variant to phenotype via SORT1 at the 1p13 cholesterol locus. Nature *466*, 714–719.
5. Cowper-Sal lari, R., Zhang, X., Wright, J.B., Bailey, S.D., Cole, M.D., Eeckhoute, J., Moore, J.H., and Lupien, M. (2012). Breast cancer risk-associated SNPs modulate the affinity of chromatin for FOXA1 and alter gene expression. Nat. Genet. *44*, 1191–1198.
6. Harismendy, O., Notani, D., Song, X., Rahim, N.G., Tanasa, B., Heintzman, N., Ren, B., Fu, X.D., Topol, E.J., Rosenfeld, M.G., and Frazer, K.A. (2011). 9p21 DNA variants associated with coronary artery disease impair interferon-γ signalling response. Nature *470*, 264–268.
7. Karczewski, K.J., Dudley, J.T., Kukurba, K.R., Chen, R., Butte, A.J., Montgomery, S.B., and Snyder, M. (2013). Systematic functional regulatory assessment of disease-associated variants. Proc. Natl. Acad. Sci. USA *110*, 9607–9612.
8. Liu, J.Z., Almarri, M.A., Gaffney, D.J., Mells, G.F., Jostins, L., Cordell, H.J., Ducker, S.J., Day, D.B., Heneghan, M.A., Neuberger, J.M., et al.; UK Primary Biliary Cirrhosis (PBC) Consortium; Wellcome Trust Case Control Consortium 3 (2012). Dense fine-mapping study identifies new susceptibility loci for primary biliary cirrhosis. Nat. Genet. *44*, 1137–1141.
9. Pasquali, L., Gaulton, K.J., Rodríguez-Seguí, S.A., Mularoni, L., Miguel-Escalada, I., Akerman, I., Tena, J.J., Morán, I., Gómez-Marín, C., van de Bunt, M., et al. (2014). Pancreatic islet enhancer clusters enriched in type 2 diabetes risk-associated variants. Nat. Genet. *46*, 136–143.
10. Paul, D.S., Albers, C.A., Rendon, A., Voss, K., Stephens, J., van der Harst, P., Chambers, J.C., Soranzo, N., Ouwehand, W.H., and Deloukas, P.; HaemGen Consortium (2013). Maps of

open chromatin highlight cell type-restricted patterns of regulatory sequence variation at hematological trait loci. Genome Res. *23*, 1130–1141.
11. Pickrell, J.K. (2014). Joint analysis of functional genomic data and genome-wide association studies of 18 human traits. Am. J. Hum. Genet. *94*, 559–573.
12. Schaub, M.A., Boyle, A.P., Kundaje, A., Batzoglou, S., and Snyder, M. (2012). Linking disease associations with regulatory information in the human genome. Genome Res. *22*, 1748–1759.
13. Dunham, I., Kundaje, A., Aldred, S.F., Collins, P.J., Davis, C.A., Doyle, F., Epstein, C.B., Frietze, S., Harrow, J., Kaul, R., et al.; ENCODE Project Consortium (2012). An integrated encyclopedia of DNA elements in the human genome. Nature *489*, 57–74.
14. Bernstein, B.E., Stamatoyannopoulos, J.A., Costello, J.F., Ren, B., Milosavljevic, A., Meissner, A., Kellis, M., Marra, M.A., Beaudet, A.L., Ecker, J.R., et al. (2010). The NIH Roadmap Epigenomics Mapping Consortium. Nat. Biotechnol. *28*, 1045–1048.
15. Adams, D., Altucci, L., Antonarakis, S.E., Ballesteros, J., Beck, S., Bird, A., Bock, C., Boehm, B., Campo, E., Caricasole, A., et al. (2012). BLUEPRINT to decode the epigenetic signature written in blood. Nat. Biotechnol. *30*, 224–226.
16. Mercer, T.R., Edwards, S.L., Clark, M.B., Neph, S.J., Wang, H., Stergachis, A.B., John, S., Sandstrom, R., Li, G., Sandhu, K.S., et al. (2013). DNase I-hypersensitive exons colocalize with promoters and distal regulatory elements. Nat. Genet. *45*, 852–859.
17. Birnbaum, R.Y., Clowney, E.J., Agamy, O., Kim, M.J., Zhao, J., Yamanaka, T., Pappalardo, Z., Clarke, S.L., Wenger, A.M., Nguyen, L., et al. (2012). Coding exons function as tissue-specific enhancers of nearby genes. Genome Res. *22*, 1059–1068.
18. Bickel, P.J., Boley, N., Brown, J.B., Huang, H., and Zhang, N.R. (2010). Subsampling methods for genomic inference. Ann. Appl. Stat. *4*, 1660–1697.
19. Khurana, E., Fu, Y., Colonna, V., Mu, X.J., Kang, H.M., Lappalainen, T., Sboner, A., Lochovsky, L., Chen, J., Harmanci, A., et al.; 1000 Genomes Project Consortium (2013). Integrative annotation of variants from 1092 humans: application to cancer genomics. Science *342*, 1235587.
20. Sankararaman, S., Mallick, S., Dannemann, M., Prüfer, K., Kelso, J., Pääbo, S., Patterson, N., and Reich, D. (2014). The genomic landscape of Neanderthal ancestry in present-day humans. Nature *507*, 354–357.
21. Abecasis, G.R., Auton, A., Brooks, L.D., DePristo, M.A., Durbin, R.M., Handsaker, R.E., Kang, H.M., Marth, G.T., and McVean, G.A.; 1000 Genomes Project Consortium (2012). An integrated map of genetic variation from 1,092 human genomes. Nature *491*, 56–65.
22. Zhang, Y., Liu, T., Meyer, C.A., Eeckhoute, J., Johnson, D.S., Bernstein, B.E., Nusbaum, C., Myers, R.M., Brown, M., Li, W., and Liu, X.S. (2008). Model-based analysis of ChIP-Seq (MACS). Genome Biol. *9*, R137.
23. Raychaudhuri, S., Plenge, R.M., Rossin, E.J., Ng, A.C., Purcell, S.M., Sklar, P., Scolnick, E.M., Xavier, R.J., Altshuler, D., and Daly, M.J.; International Schizophrenia Consortium (2009). Identifying relationships among genomic disease regions: predicting genes at pathogenic SNP associations and rare deletions. PLoS Genet. *5*, e1000534.
24. Ng, P.C., and Henikoff, S. (2003). SIFT: Predicting amino acid changes that affect protein function. Nucleic Acids Res. *31*, 3812–3814.
25. Welter, D., MacArthur, J., Morales, J., Burdett, T., Hall, P., Junkins, H., Klemm, A., Flicek, P., Manolio, T., Hindorff, L., and Parkinson, H. (2014). The NHGRI GWAS Catalog, a curated resource of SNP-trait associations. Nucleic Acids Res. *42*, D1001–D1006.
26. Wood, A.R., Esko, T., Yang, J., Vedantam, S., Pers, T.H., Gustafsson, S., Chu, A.Y., Estrada, K., Luan, J., Kutalik, Z., et al.; Electronic Medical Records and Genomics (eMEMERGEGE) Consortium; MIGen Consortium; PAGEGE Consortium; LifeLines Cohort Study (2014). Defining the role of common variation in the genomic and biological architecture of adult human height. Nat. Genet. *46*, 1173–1186.
27. Okada, Y., Wu, D., Trynka, G., Raj, T., Terao, C., Ikari, K., Kochi, Y., Ohmura, K., Suzuki, A., Yoshida, S., et al.; RACI consortium; GARNET consortium (2014). Genetics of rheumatoid arthritis contributes to biology and drug discovery. Nature *506*, 376–381.
28. Michailidou, K., Hall, P., Gonzalez-Neira, A., Ghoussaini, M., Dennis, J., Milne, R.L., Schmidt, M.K., Chang-Claude, J., Bojesen, S.E., Bolla, M.K., et al.; Breast and Ovarian Cancer Susceptibility Collaboration; Hereditary Breast and Ovarian Cancer Research Group Netherlands (HEBON); kConFab Investigators; Australian Ovarian Cancer Study Group; GENICA (Gene Environment Interaction and Breast Cancer in Germany) Network (2013). Large-scale genotyping identifies 41 new loci associated with breast cancer risk. Nat. Genet. *45*, 353–361.e1–e2.
29. Westra, H.J., Peters, M.J., Esko, T., Yaghootkar, H., Schurmann, C., Kettunen, J., Christiansen, M.W., Fairfax, B.P., Schramm, K., Powell, J.E., et al. (2013). Systematic identification of trans eQTLs as putative drivers of known disease associations. Nat. Genet. *45*, 1238–1243.
30. Purcell, S., Neale, B., Todd-Brown, K., Thomas, L., Ferreira, M.A., Bender, D., Maller, J., Sklar, P., de Bakker, P.I., Daly, M.J., and Sham, P.C. (2007). PLINK: a tool set for whole-genome association and population-based linkage analyses. Am. J. Hum. Genet. *81*, 559–575.
31. Gerasimova, A., Chavez, L., Li, B., Seumois, G., Greenbaum, J., Rao, A., Vijayanand, P., and Peters, B. (2013). Predicting cell types and genetic variations contributing to disease by combining GWAS and epigenetic data. PLoS ONE *8*, e54359.
32. Lee, Y., Gamazon, E.R., Rebman, E., Lee, Y., Lee, S., Dolan, M.E., Cox, N.J., and Lussier, Y.A. (2012). Variants affecting exon skipping contribute to complex traits. PLoS Genet. *8*, e1002998.
33. Anttila, V., Winsvold, B.S., Gormley, P., Kurth, T., Bettella, F., McMahon, G., Kallela, M., Malik, R., de Vries, B., Terwindt, G., et al.; North American Brain Expression Consortium; UK Brain Expression Consortium; International Headache Genetics Consortium (2013). Genome-wide meta-analysis identifies new susceptibility loci for migraine. Nat. Genet. *45*, 912–917.
34. Willer, C.J., Schmidt, E.M., Sengupta, S., Peloso, G.M., Gustafsson, S., Kanoni, S., Ganna, A., Chen, J., Buchkovich, M.L., Mora, S., et al.; Global Lipids Genetics Consortium (2013). Discovery and refinement of loci associated with lipid levels. Nat. Genet. *45*, 1274–1283.
35. Lappalainen, T., Sammeth, M., Friedländer, M.R., ’t Hoen, P.A., Monlong, J., Rivas, M.A., Gonzàlez-Porta, M., Kurbatova, N., Griebel, T., Ferreira, P.G., et al.; Geuvadis Consortium (2013). Transcriptome and genome sequencing uncovers functional variation in humans. Nature *501*, 506–511.
36. Akhtar-Zaidi, B., Cowper-Sal-lari, R., Corradin, O., Saiakhova, A., Bartels, C.F., Balasubramanian, D., Myeroff, L., Lutterbaugh,

J., Jarrar, A., Kalady, M.F., et al. (2012). Epigenomic enhancer profiling defines a signature of colon cancer. Science *336*, 736–739.

37. Corradin, O., Saiakhova, A., Akhtar-Zaidi, B., Myeroff, L., Willis, J., Cowper-Sal lari, R., Lupien, M., Markowitz, S., and Scacheri, P.C. (2014). Combinatorial effects of multiple enhancer variants in linkage disequilibrium dictate levels of gene expression to confer susceptibility to common traits. Genome Res. *24*, 1–13.
38. Ernst, J., Kheradpour, P., Mikkelsen, T.S., Shoresh, N., Ward, L.D., Epstein, C.B., Zhang, X., Wang, L., Issner, R., Coyne, M., et al. (2011). Mapping and analysis of chromatin state dynamics in nine human cell types. Nature *473*, 43–49.
39. Brown, C.D., Mangravite, L.M., and Engelhardt, B.E. (2013). Integrative modeling of eQTLs and cis-regulatory elements suggests mechanisms underlying cell type specificity of eQTLs. PLoS Genet. *9*, e1003649.
40. Degner, J.F., Pai, A.A., Pique-Regi, R., Veyrieras, J.B., Gaffney, D.J., Pickrell, J.K., De Leon, S., Michelini, K., Lewellen, N., Crawford, G.E., et al. (2012). DNase I sensitivity QTLs are a major determinant of human expression variation. Nature *482*, 390–394.
41. Gaffney, D.J., Veyrieras, J.B., Degner, J.F., Pique-Regi, R., Pai, A.A., Crawford, G.E., Stephens, M., Gilad, Y., and Pritchard, J.K. (2012). Dissecting the regulatory architecture of gene expression QTLs. Genome Biol. *13*, R7.
42. Stranger, B.E., Forrest, M.S., Dunning, M., Ingle, C.E., Beazley, C., Thorne, N., Redon, R., Bird, C.P., de Grassi, A., Lee, C., et al. (2007). Relative impact of nucleotide and copy number variation on gene expression phenotypes. Science *315*, 848–853.
43. Veyrieras, J.B., Kudaravalli, S., Kim, S.Y., Dermitzakis, E.T., Gilad, Y., Stephens, M., and Pritchard, J.K. (2008). High-resolution mapping of expression-QTLs yields insight into human gene regulation. PLoS Genet. *4*, e1000214.
44. Barreau, C., Paillard, L., and Osborne, H.B. (2005). AU-rich elements and associated factors: are there unifying principles? Nucleic Acids Res. *33*, 7138–7150.
45. Pasquinelli, A.E. (2012). MicroRNAs and their targets: recognition, regulation and an emerging reciprocal relationship. Nat. Rev. Genet. *13*, 271–282.
46. Sandberg, R., Neilson, J.R., Sarma, A., Sharp, P.A., and Burge, C.B. (2008). Proliferating cells express mRNAs with shortened 3′ untranslated regions and fewer microRNA target sites. Science *320*, 1643–1647.
47. Hu, X., Kim, H., Raj, T., Brennan, P.J., Trynka, G., Teslovich, N., Slowikowski, K., Chen, W.M., Onengut, S., Baecher-Allan, C., et al. (2014). Regulation of gene expression in autoimmune disease loci and the genetic basis of proliferation in CD4+ effector memory T cells. PLoS Genet. *10*, e1004404.
48. Hu, X., Kim, H., Stahl, E., Plenge, R., Daly, M., and Raychaudhuri, S. (2011). Integrating autoimmune risk loci with gene-expression data identifies specific pathogenic immune cell subsets. Am. J. Hum. Genet. *89*, 496–506.
49. Pepke, S., Wold, B., and Mortazavi, A. (2009). Computation for ChIP-seq and RNA-seq studies. Nat. Methods *6* (11, Suppl), S22–S32.
50. Ward, L.D., and Kellis, M. (2012). HaploReg: a resource for exploring chromatin states, conservation, and regulatory motif alterations within sets of genetically linked variants. Nucleic Acids Res. *40*, D930–D934.
51. Folkerd, E.J., and Dowsett, M. (2010). Influence of sex hormones on cancer progression. J. Clin. Oncol. *28*, 4038–4044.
52. Jordan, V.C. (2004). Selective estrogen receptor modulation: concept and consequences in cancer. Cancer Cell *5*, 207–213.
53. McDonald, T.J., and Ellard, S. (2013). Maturity onset diabetes of the young: identification and diagnosis. Ann. Clin. Biochem. *50*, 403–415.
54. Santangelo, L., Marchetti, A., Cicchini, C., Conigliaro, A., Conti, B., Mancone, C., Bonzo, J.A., Gonzalez, F.J., Alonzi, T., Amicone, L., and Tripodi, M. (2011). The stable repression of mesenchymal program is required for hepatocyte identity: a novel role for hepatocyte nuclear factor 4α. Hepatology *53*, 2063–2074.
55. DeLaForest, A., Nagaoka, M., Si-Tayeb, K., Noto, F.K., Konopka, G., Battle, M.A., and Duncan, S.A. (2011). HNF4A is essential for specification of hepatic progenitors from human pluripotent stem cells. Development *138*, 4143–4153.
56. Vahedi, G., Kanno, Y., Furumoto, Y., Jiang, K., Parker, S.C., Erdos, M.R., Davis, S.R., Roychoudhuri, R., Restifo, N.P., Gadina, M., et al. (2015). Super-enhancers delineate disease-associated regulatory nodes in T cells. Nature *520*, 558–562.
57. de Bakker, P.I., and Raychaudhuri, S. (2012). Interrogating the major histocompatibility complex with high-throughput genomics. Hum. Mol. Genet. *21* (R1), R29–R36.
58. Graham, R.R., Kozyrev, S.V., Baechler, E.C., Reddy, M.V., Plenge, R.M., Bauer, J.W., Ortmann, W.A., Koeuth, T., González Escribano, M.F., Pons-Estel, B., et al.; Argentine and Spanish Collaborative Groups (2006). A common haplotype of interferon regulatory factor 5 (IRF5) regulates splicing and expression and is associated with increased risk of systemic lupus erythematosus. Nat. Genet. *38*, 550–555.
59. Maller, J.B., McVean, G., Byrnes, J., Vukcevic, D., Palin, K., Su, Z., Howson, J.M., Auton, A., Myers, S., Morris, A., et al.; Wellcome Trust Case Control Consortium (2012). Bayesian refinement of association signals for 14 loci in 3 common diseases. Nat. Genet. *44*, 1294–1301.
60. Forrest, A.R., Kawaji, H., Rehli, M., Baillie, J.K., de Hoon, M.J., Haberle, V., Lassmann, T., Kulakovskiy, I.V., Lizio, M., Itoh, M., et al.; FANTOM Consortium and the RIKEN PMI and CLST (DGT) (2014). A promoter-level mammalian expression atlas. Nature *507*, 462–470.

The American Journal of Human Genetics

Supplemental Data

Disentangling the Effects of Colocalizing Genomic Annotations to Functionally Prioritize Non-coding Variants within Complex-Trait Loci

Gosia Trynka, Harm-Jan Westra, Kamil Slowikowski, Xinli Hu, Han Xu, Barbara E Stranger, Robert J Klein, Buhm Han, and Soumya Raychaudhuri

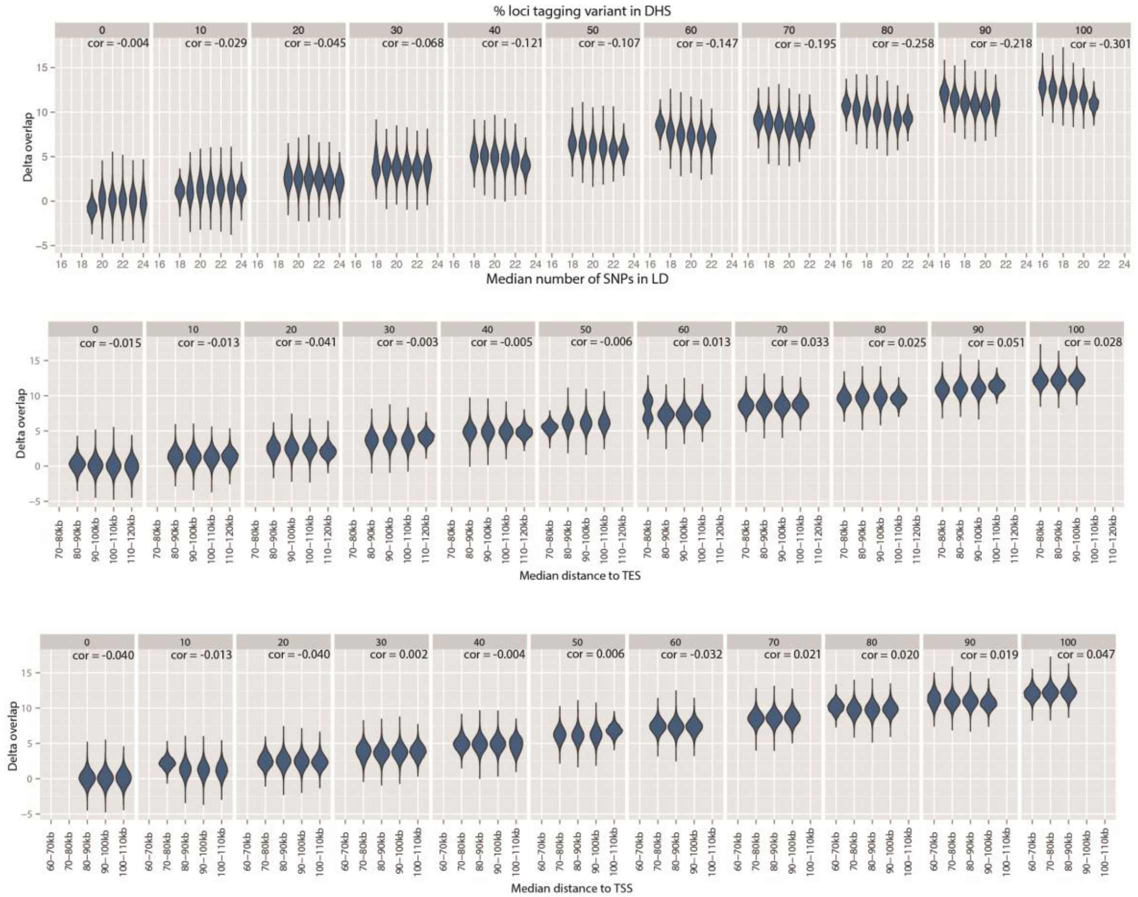

Figure S1. Influence of different genomic parameters on delta-overlap. For each of the 1,000 simulated SNP sets tagging variable percent of DHS variants, we assessed if SNP structure (measured by the number of LD SNPs) or genomic features, such as proximity to TSS and TES, could influence the estimates of delta-overlap. For each SNP set, we plot the median characteristics and the set and the delta overlap. While we observed that the proportion of causal variants within DHS regions was proportional with the delta-overlap, for a fixed proportion of causal DHS variants delta-overlap is stable for all genomic features (r^2<0.1). We observed weak correlation with the number of LD SNPs in SNP sets that were highly saturated with DHS-tagging loci (> 60%).

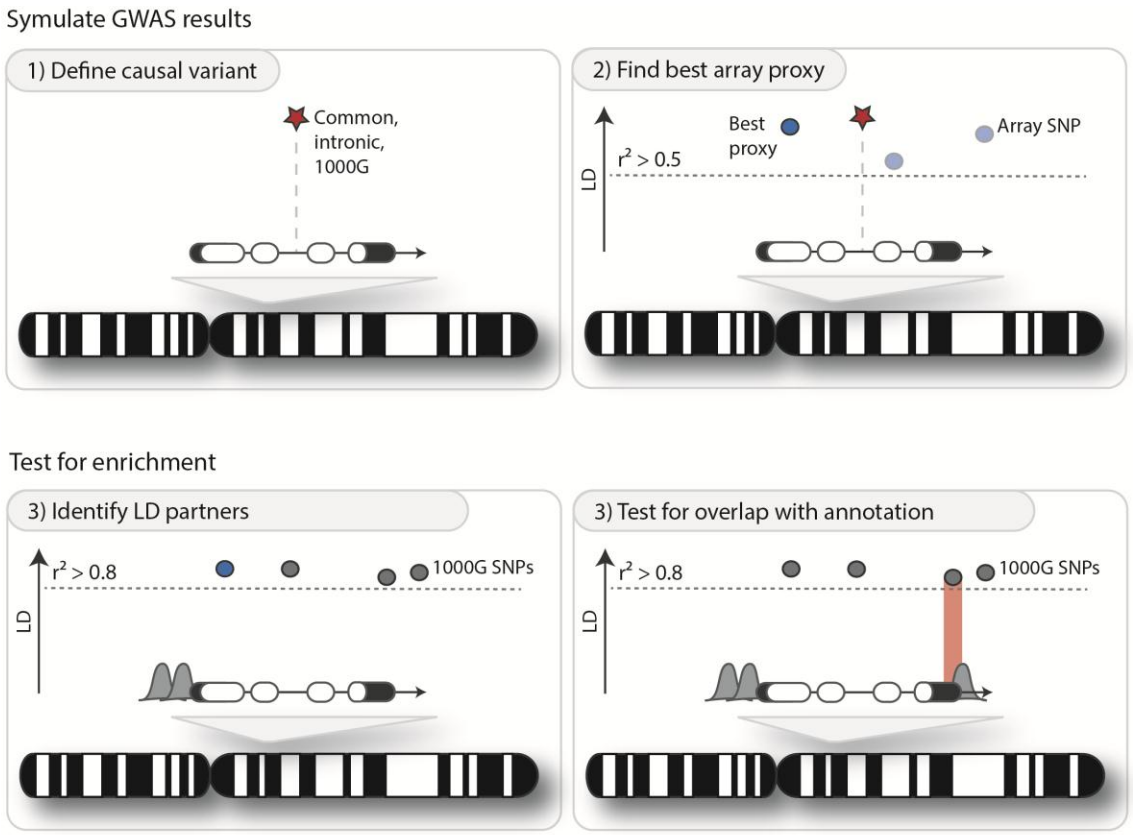

Figure S2. Schematic figure of the strategy to simulate the GWAS SNP sets. From 1000 Genomes common European variants, we pick a functional SNP that maps within a pre-defined genomic annotation, e.g. intron. To imitate a GWAS approach we then identify the SNP on the genotyping array (Illumina Human Omni2.5 chip) that best tags (r^2>0.5) the selected predefined functional variant. We construct sets of 1,416 SNPs tagging predefined functional variants. These sets are then subject to enrichment tests.

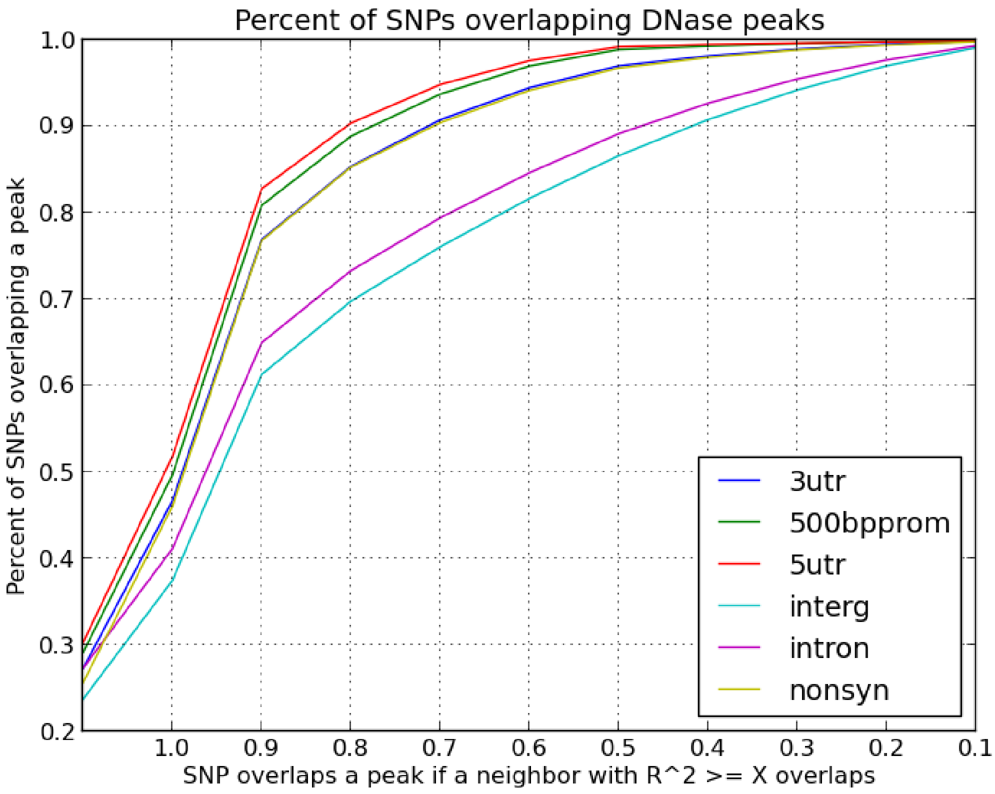

Figure S3. Quantification of measured overlap for Illumina Omni2.5 SNPs tagging functional variants from different annotations. About 30% of SNPs at Illumina Omni2.5 overlap with DHS sites on their own. This percentage quickly increases as additional linked SNPs are included. At r^2=0.8 we should observe the strongest enrichment for promoter and 5'UTR SNPs, moderate signals for coding (non-synonymous) and 3'UTR, and no enrichment for intron or intergenic regions. The decrease in r^2 threshold increases the percentage of SNPs overlapping with DHS. Importantly, when deriving the null through matching-based tests, not accounting for the number of LD SNP dramatically affects the enrichment results.

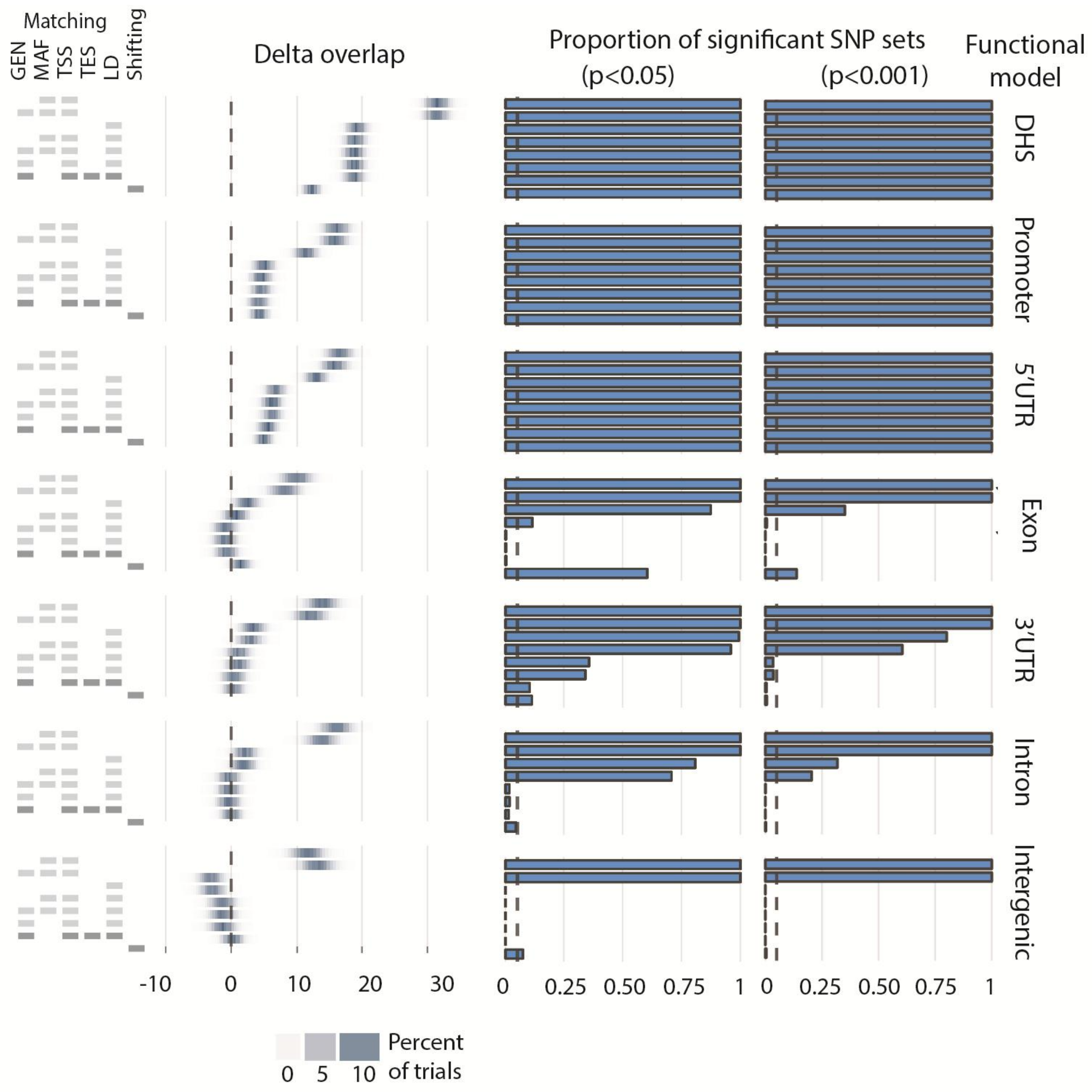

Figure S4. **Comparison of the effect sizes (delta-overlap) and power of different enrichment methods.** Not matching on the number of SNPs in LD results in global inflation in statistics. For example, we observed p<0.001 significance across all regulatory and non-regulatory SNP sets. However, matching on LD alone is insufficient; we observed consistently inflated type I error across SNP sets tagging functional variants from introns (p<0.05 in 81% of 1,000 SNP sets). We also observed that the standard matching parameters were inadequate across SNP sets that tagged variants in 3'UTR and exons. Accounting for the distance to the end of transcription (TES) was crucial if functional variants were selected from the 3'UTR, and it decreased the false positive rate by 26% (at p<0.05). Note that including MAF in the matching parameters has no effect, even though it is a frequently used parameter in SNP matching-based tests.

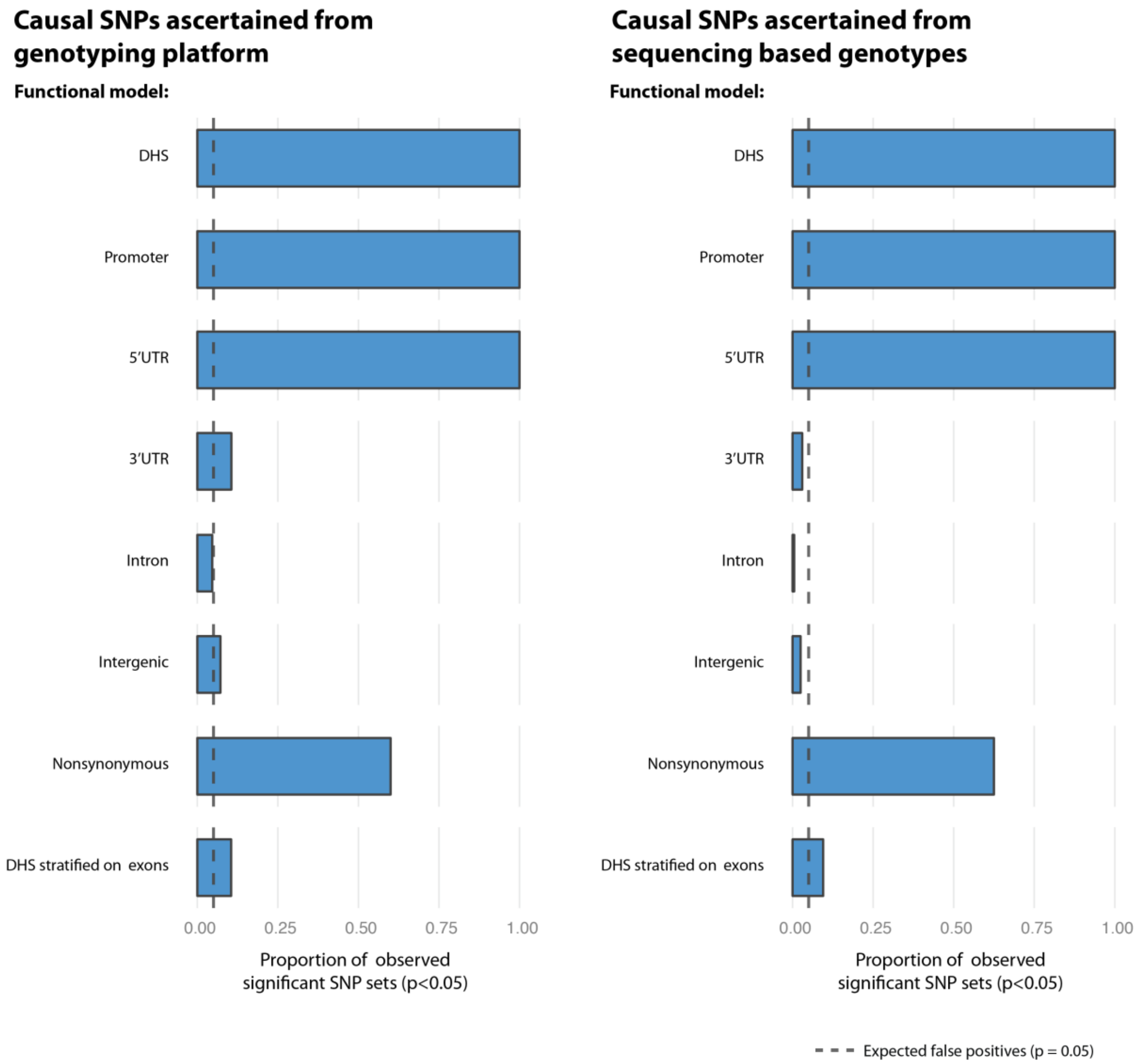

Figure S5. Performance of *GoShifter* when selecting tag variants from sequencing data. We generated sets of 1,416 SNPs that tag functional variants from SNPs within annotations enriched for DHS (SNPs overlapping DHS, promoter regions, 5' UTR, nonsynonymous variants in exons) or depleted for DHS (3'UTR, introns and intergenic regions). We generated 1,000 sets of SNPs where we selected tagging SNPs from a commercial genotyping platform (left), and 200 sets of tagging SNPs were selected from a sequencing based study (1000 Genomes Project, right). Array-based data is identical to the data presented in Figure 2. Then we subsequently tested for enrichment for DHS with GoShifter. We also tested the number of SNP sets obtaining $p<0.05$ stratifying DHS for sets where causal variants were in exons. The proportion of expected sets $p<0.05$ under the null is indicated by the dotted line.

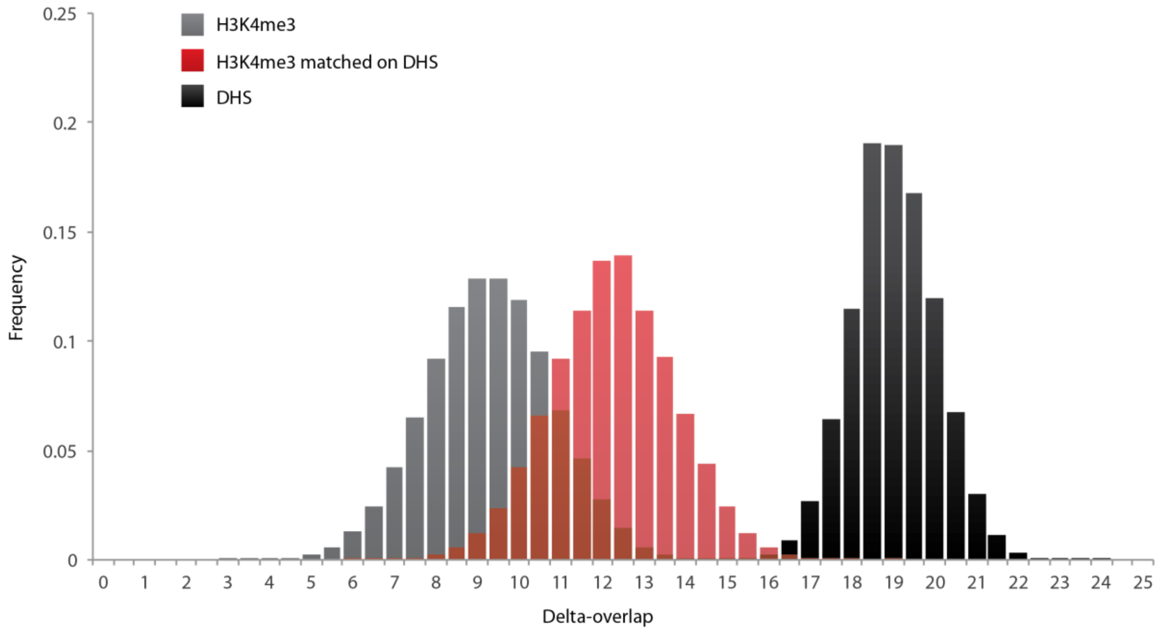

Figure S6. Conditional matching and colocalizing annotations. We assessed the feasibility of using a matching based approach to disentangle colocalizing annotations. For this purpose, we generated 1,000 sets of SNPs tagging functional variants in DHS. We tested these sets for enrichment with DHS (black) and the colocalizing annotation H3K4me3 (grey) using matching based on (GEN, TSS, TES and LD). To account for colocalization, we appended an extra matching parameter (DHS) and repeated the H3K4me3 enrichment analysis (red). We found that each of these enrichment analyses yielded significant enrichment ($p<0.05$) for 100% of the generated sets (as indicated by the delta-overlap values being all greater than zero). Matching on the additional DHS parameter did not mitigate the delta-overlap for H3K4me3 enrichment. This is probably due to confounding factors included by the extra DHS matching parameter (for example LD bias within DHS overlapping variants), and the lack of correlation between the associated and causal SNP in the annotations they occur in.

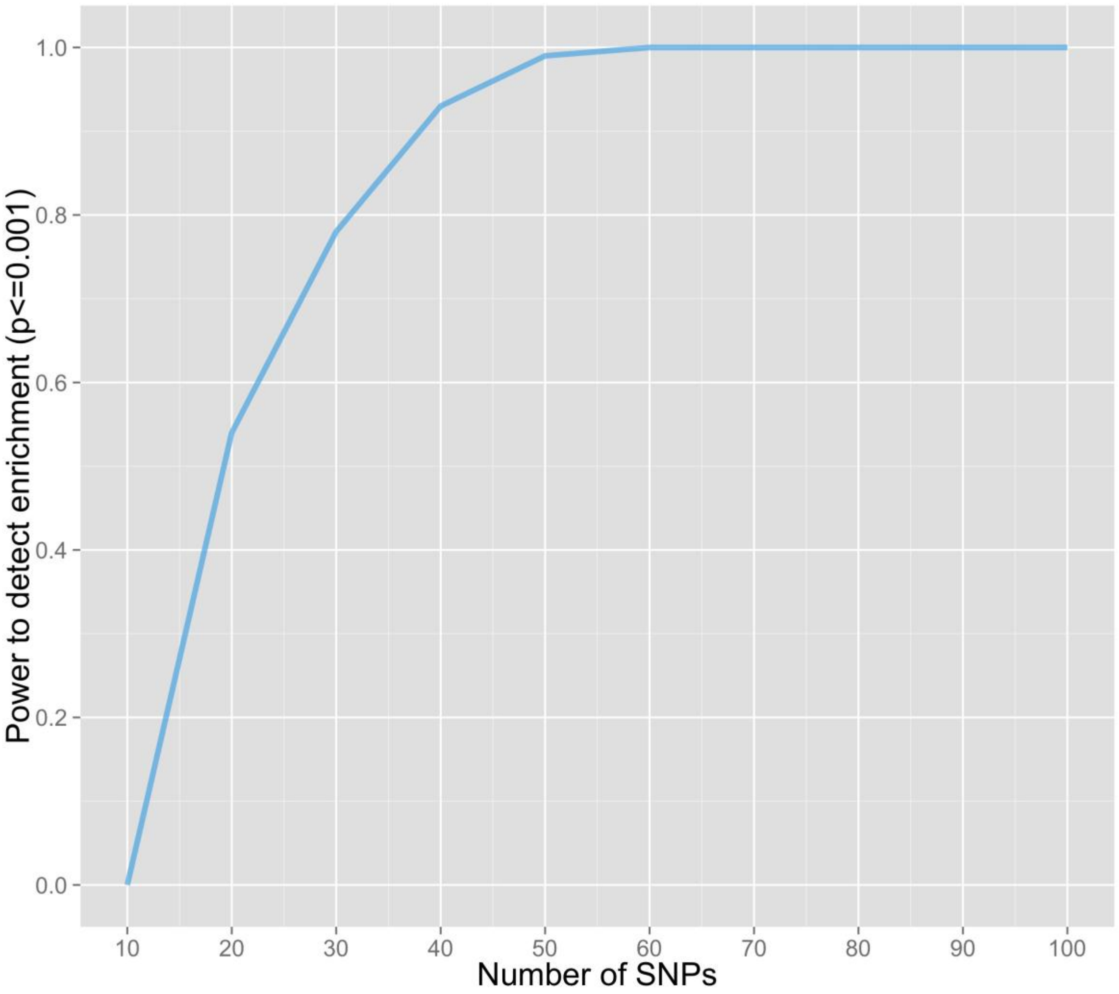

Figure S7. Power to detect significant enrichment as a function of the number of tested SNPs. We subsampled SNPs from SNP sets tagging variants in DHS regions (Figure 2A and S4 – functional model with DHS variants). To obtain power estimates for *GoShifter* to detect significant enrichment we generated a hundred SNP sets with variable number of variants (from 10 to 100 incremented by 10 SNPs). With 30 SNPs *GoShifter* has 80% of power to detect significant enrichment ($p<0.05$).

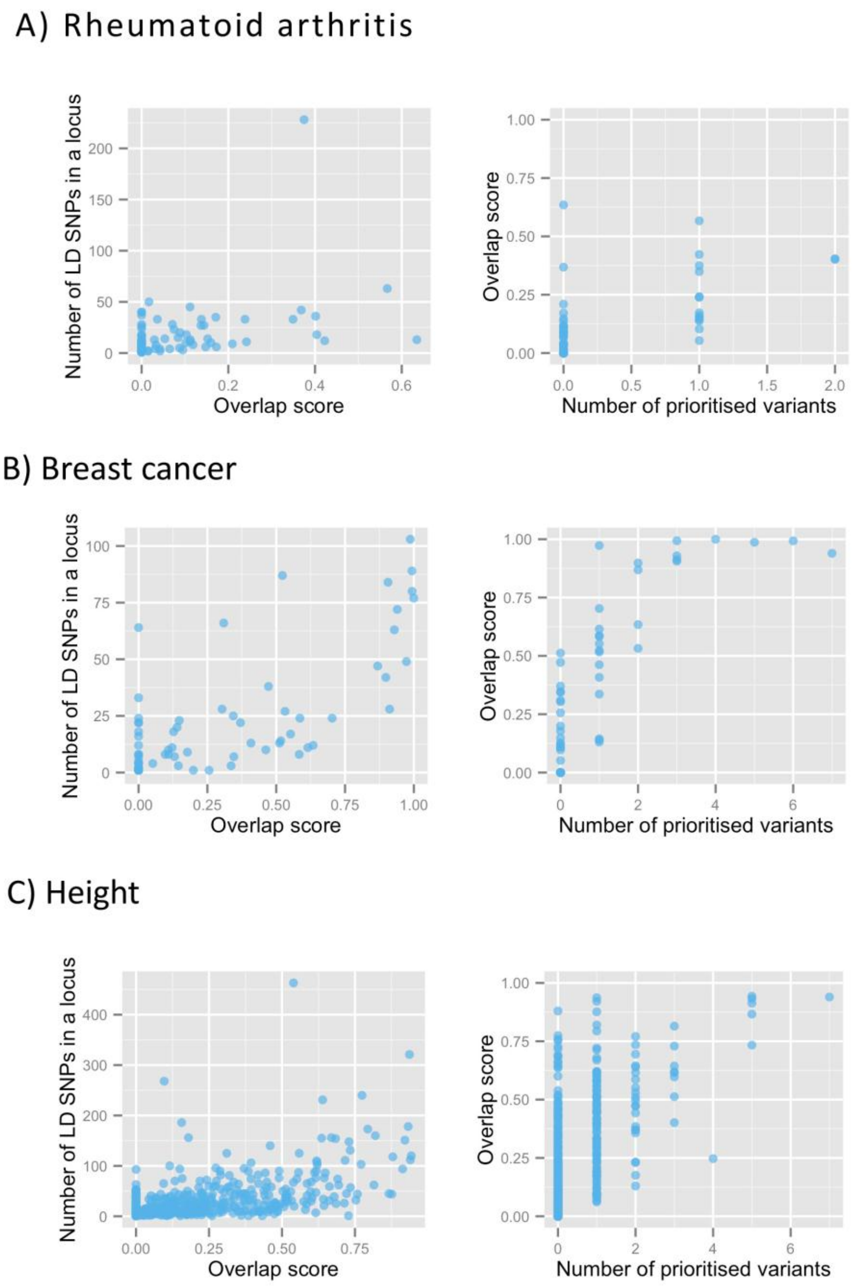

Figure S8. Prioritization of functional variants using overlap score. Here we define a prioritized variant, as a variant(s) within a locus overlapping the annotation in question. For each tested trait we plot the relationship of the overlap score of each locus and the number of SNPs in LD within each associated locus (left). We also plotted the relationship of the overlap score and the number of those SNPs in LD overlapping an annotation site (e.g. prioritized variants).

Table S1. Overview of published enrichment methods. Published statistical strategies within the literature to assess enrichment for different annotations.

Trait	Annotation	Enrichment method	Reference
NHGRI GWAS Catalog	TFs ChIP-seq, DHS	Matching on MAF, TSS, platform, UCSC gene predicted function	[1]
144 diseases	NF-kB ChIP-seq	Matching on MAF, TSS	[2]
Platelet and erythrocyte phenotypes	FAIRE-seq	Matching on MAF, TSS and LD	[3]
Breast cancer	TF and histone modification ChIP-seq	LD	[4]
Primary biliary cirrhosis	DHS, FAIRE-seq	Permutations with SNPs within associated loci	[5]
Asthma	Chromatin HMM states, H3K4me1, H3K27ac	None	[6]
NHGRI GWAS Catalog	DHS	MAF, TSS, GENIC	[7]
NHGRI GWAS Catalog	Intronic splicing enhancers	MAF, TSS	[8]
NHGRI GWAS Catalog - trans-eQTL SNPs	CNVs, TFBS, splice enhancers/silencers, histone enhancers	Relative to disease associated non-trans-eQTL SNPs	[9]
NHGRI GWAS Catalog	DHS, TFBS	Matching on MAF, TSS, genomic location	[10]
Migraine	DHS	MAF, TSS, GC content	[11]
Lipid levels	Chromatin HMM states, histone modifications, open chromatin	MAF, TSS, LD partners	[12]
eQTLs	Histone modifications	MAF, TSS	[13]
Colorectal cancer SNPs	Enhancers	LD, IlluminOmniExpress	[14]
Immune-related disease SNPs	Enhancers	LD, IlluminOmniExpress	[15]
Specific traits from GWAS Catalog; T2D and fasting glycemia	Human pancreatic islet TFBS and enhancer clusters	LD; TSS, MAF	[16]
GWAS Catalog traits	Chromatin states	Shifts and permutations within GWAS Catalog traits	[17]
Rare variants and structural variations (SVs)/SNPs/eQTLs	GENECODE annotation/functional annotations	Annotation shifting/MAF+TSS matching	[18]

Table S2. Detailed description of used cell types for DHS, H3K4me1, H3K4me3, and H3K9ac.

Table S3. Proportion of 1,000 SNP sets, derived from causal variants with specific functional annotations, demonstrating enrichment at p<0.05.

Enrichment method	Promoter	5'UTR	Non-synonymous	3'UTR	Intron	Intergenic
**GEN+TSS+TES+LD**	1	1	0.002	0.102	0.013	0

**GEN+MAF+TSS+LD**	1	1	0.001	0.358	0.016	0
**GEN+MAF+TSS**	1	1	1	1	1	1
**GEN+TSS+LD**	1	1	0.001	0.342	0.017	0
**MAF+TSS+LD**	1	1	0.114	0.959	0.708	0
**MAF+TSS**	1	1	1	1	1	1
**LD**	1	1	0.875	0.994	0.808	0
**Local shifts**	1	1	0.604	0.112	0.044	0.074

References

1. Schaub, M.A., Boyle, A.P., Kundaje, A., Batzoglou, S., and Snyder, M. (2012). Linking disease associations with regulatory information in the human genome. Genome research 22, 1748-1759.
2. Karczewski, K.J., Dudley, J.T., Kukurba, K.R., Chen, R., Butte, A.J., Montgomery, S.B., and Snyder, M. (2013). Systematic functional regulatory assessment of disease-associated variants. Proceedings of the National Academy of Sciences of the United States of America 110, 9607-9612.
3. Paul, D.S., Albers, C.A., Rendon, A., Voss, K., Stephens, J., van der Harst, P., Chambers, J.C., Soranzo, N., Ouwehand, W.H., and Deloukas, P. (2013). Maps of open chromatin highlight cell type-restricted patterns of regulatory sequence variation at hematological trait loci. Genome research 23, 1130-1141.
4. Cowper-Sal lari, R., Zhang, X., Wright, J.B., Bailey, S.D., Cole, M.D., Eeckhoute, J., Moore, J.H., and Lupien, M. (2012). Breast cancer risk-associated SNPs modulate the affinity of chromatin for FOXA1 and alter gene expression. Nature genetics 44, 1191-1198.
5. Liu, J.Z., Almarri, M.A., Gaffney, D.J., Mells, G.F., Jostins, L., Cordell, H.J., Ducker, S.J., Day, D.B., Heneghan, M.A., Neuberger, J.M., et al. (2012). Dense fine-mapping study identifies new susceptibility loci for primary biliary cirrhosis. Nature genetics 44, 1137-1141.
6. Gerasimova, A., Chavez, L., Li, B., Seumois, G., Greenbaum, J., Rao, A., Vijayanand, P., and Peters, B. (2013). Predicting cell types and genetic variations contributing to disease by combining GWAS and epigenetic data. PloS one 8, e54359.
7. Maurano, M.T., Humbert, R., Rynes, E., Thurman, R.E., Haugen, E., Wang, H., Reynolds, A.P., Sandstrom, R., Qu, H., Brody, J., et al. (2012). Systematic localization of common disease-associated variation in regulatory DNA. Science 337, 1190-1195.
8. Lee, Y., Gamazon, E.R., Rebman, E., Lee, Y., Lee, S., Dolan, M.E., Cox, N.J., and Lussier, Y.A. (2012). Variants affecting exon skipping contribute to complex traits. PLoS genetics 8, e1002998.
9. Westra, H.J., Peters, M.J., Esko, T., Yaghootkar, H., Schurmann, C., Kettunen, J., Christiansen, M.W., Fairfax, B.P., Schramm, K., Powell, J.E., et al. (2013). Systematic identification of trans eQTLs as putative drivers of known disease associations. Nature genetics.
10. Dunham, I., Kundaje, A., Aldred, S.F., Collins, P.J., Davis, C.A., Doyle, F., Epstein, C.B., Frietze, S., Harrow, J., Kaul, R., et al. (2012). An integrated encyclopedia of DNA elements in the human genome. Nature 489, 57-74.
11. Anttila, V., Winsvold, B.S., Gormley, P., Kurth, T., Bettella, F., McMahon, G., Kallela, M., Malik, R., de Vries, B., Terwindt, G., et al. (2013). Genome-wide meta-analysis identifies new susceptibility loci for migraine. Nature genetics 45, 912-917.
12. Willer, C.J., Schmidt, E.M., Sengupta, S., Peloso, G.M., Gustafsson, S., Kanoni, S., Ganna, A., Chen, J., Buchkovich, M.L., Mora, S., et al. (2013). Discovery and refinement of loci associated with lipid levels. Nature genetics 45, 1274-1283.
13. Lappalainen, T., Sammeth, M., Friedlander, M.R., t Hoen, P.A., Monlong, J., Rivas, M.A., Gonzalez-Porta, M., Kurbatova, N., Griebel, T., Ferreira, P.G., et al. (2013). Transcriptome and genome sequencing uncovers functional variation in humans. Nature 501, 506-511.

14. Akhtar-Zaidi, B., Cowper-Sal-lari, R., Corradin, O., Saiakhova, A., Bartels, C.F., Balasubramanian, D., Myeroff, L., Lutterbaugh, J., Jarrar, A., Kalady, M.F., et al. (2012). Epigenomic enhancer profiling defines a signature of colon cancer. Science 336, 736-739.
15. Corradin, O., Saiakhova, A., Akhtar-Zaidi, B., Myeroff, L., Willis, J., Cowper-Sal lari, R., Lupien, M., Markowitz, S., and Scacheri, P.C. (2014). Combinatorial effects of multiple enhancer variants in linkage disequilibrium dictate levels of gene expression to confer susceptibility to common traits. Genome research 24, 1-13.
16. Pasquali, L., Gaulton, K.J., Rodriguez-Segui, S.A., Mularoni, L., Miguel-Escalada, I., Akerman, I., Tena, J.J., Moran, I., Gomez-Marin, C., van de Bunt, M., et al. (2014). Pancreatic islet enhancer clusters enriched in type 2 diabetes risk-associated variants. Nature genetics 46, 136-143.
17. Ernst, J., Kheradpour, P., Mikkelsen, T.S., Shoresh, N., Ward, L.D., Epstein, C.B., Zhang, X., Wang, L., Issner, R., Coyne, M., et al. (2011). Mapping and analysis of chromatin state dynamics in nine human cell types. Nature 473, 43-49.
18. Khurana, E., Fu, Y., Colonna, V., Mu, X.J., Kang, H.M., Lappalainen, T., Sboner, A., Lochovsky, L., Chen, J., Harmanci, A., et al. (2013). Integrative annotation of variants from 1092 humans: application to cancer genomics. Science 342, 1235587.
